# Bacterial Enoyl-Reductases: The Ever-Growing List of Fabs, Their Mechanisms and Inhibition

**DOI:** 10.3389/fmicb.2022.891610

**Published:** 2022-06-16

**Authors:** Fernanda S. M. Hopf, Candida D. Roth, Eduardo V. de Souza, Luiza Galina, Alexia M. Czeczot, Pablo Machado, Luiz A. Basso, Cristiano V. Bizarro

**Affiliations:** ^1^Centro de Pesquisas em Biologia Molecular e Funcional (CPBMF) and Instituto Nacional de Ciência e Tecnologia em Tuberculose (INCT-TB), Pontifícia Universidade Católica do Rio Grande do Sul (PUCRS), Porto Alegre, Brazil; ^2^Programa de Pós-Graduação em Biologia Celular e Molecular, Pontifícia Universidade Católica do Rio Grande do Sul, Porto Alegre, Brazil; ^3^Programa de Pós-Graduação em Medicina e Ciências da Saúde, Pontifícia Universidade Católica do Rio Grande do Sul, Porto Alegre, Brazil

**Keywords:** fatty acid synthesis, FabI, FabK, FabV, Fabl, FabL2, drug targets, kinetic mechanisms

## Abstract

Enoyl-ACP reductases (ENRs) are enzymes that catalyze the last step of the elongation cycle during fatty acid synthesis. In recent years, new bacterial ENR types were discovered, some of them with structures and mechanisms that differ from the canonical bacterial FabI enzymes. Here, we briefly review the diversity of structural and catalytic properties of the canonical FabI and the new FabK, FabV, FabL, and novel ENRs identified in a soil metagenome study. We also highlight recent efforts to use the newly discovered Fabs as targets for drug development and consider the complex evolutionary history of this diverse set of bacterial ENRs.

## Introduction

**Figure 1:**
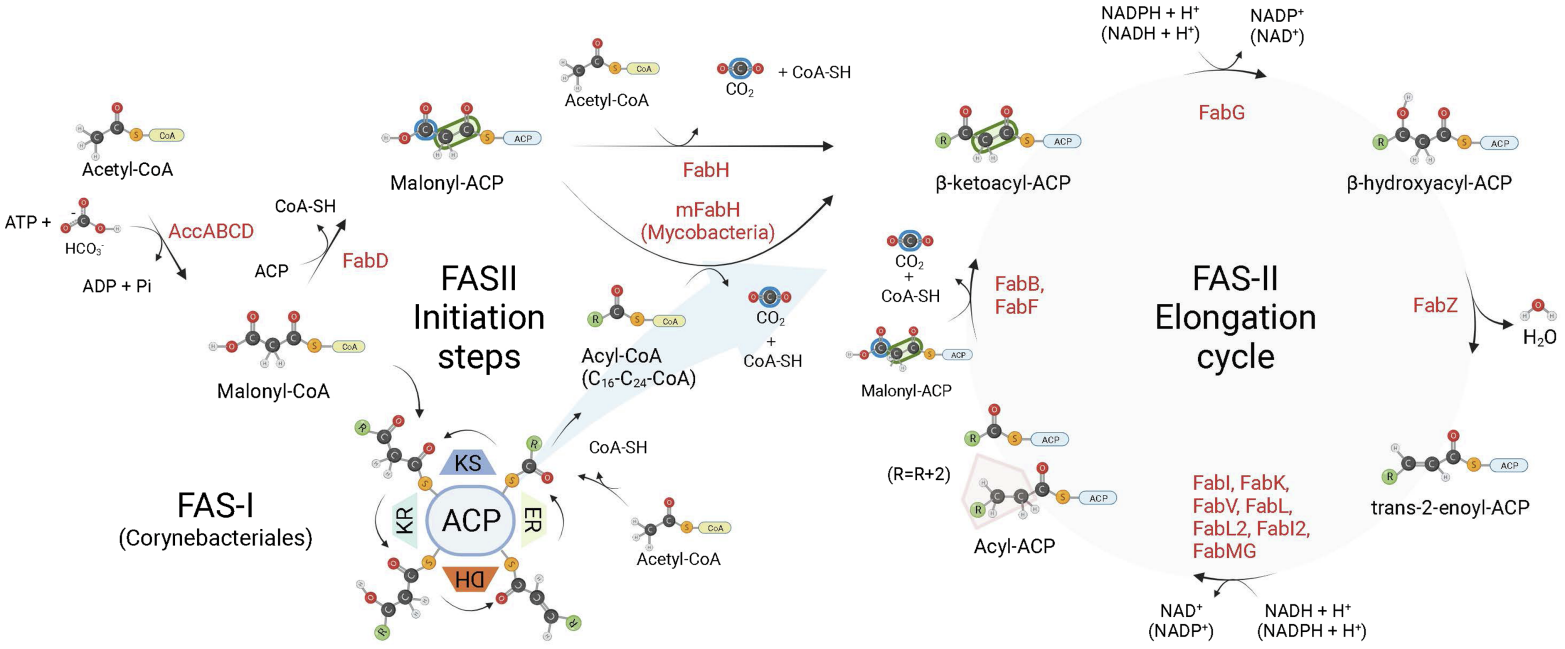
Bacterial fatty acid biosynthesis. There are two types of fatty acid (FA) biosynthesis in nature, FAS-I and FAS-II. In both pathways, fatty acid chains are elongated through iterative cycles in which two-carbon units are added after each cycle. Most bacteria rely exclusively on FAS-II pathway for FA synthesis, some members of the Corynebacteriales order, on the other hand, rely exclusively on FAS-I, while mycobacteria and most corynebacteria (mycolic acid-producing organisms) possess FAS-I and FAS-II pathways. In both systems, there are four sequential steps in each elongation cycle: (1) condensation of acyl and malonyl groups, (2) β-ketoacyl-ACP reduction, (3) β-hydroxyacyl-ACP dehydration, and (4) trans-2-enoyl-ACP reduction, leading to an extended acyl chain containing 2 additional carbon units. In FAS-I, a single megasynthase performs the four sequential steps of chain elongation, while in FAS-II different enzyme components perform each step. The first committed step in FA synthesis is the carboxylation of acetyl-CoA by the enzyme acetyl-CoA carboxylase (AccABCD), leading to the production of malonyl-CoA from acetyl-CoA. During the elongation steps of preformed acyl chains, malonyl-CoA units are the malonyl donor groups in the FAS-I condensation step (ketoacyl synthase, KS), while in FAS-II it is first converted to malonyl-ACP by FabD, which is then used by FabB (or FabF) as the malonyl donor group in the condensation step of FAS-II elongation. In FAS-I, chain initiation requires the condensation of acetyl-CoA and malonyl-CoA by the KS activity of the megasynthase. For bacteria relying exclusively on FAS-II for FA synthesis, the initiation step requires the production of acetoacetyl-ACP (a β-ketoacyl-ACP species) from the condensation of malonyl-ACP and acetyl-CoA performed by FabH. When both FAS-I and FAS-II systems are available, as in mycobacteria, preformed acyl-CoA chains containing from C16-C24 carbon units derived from the FAS-I system are shuttled into FAS-II by an initial condensation step with malonyl-ACP catalyzed by mFabH. Bacterial enoyl-ACP reductases (ENRs) catalyze the last step of trans-2-enoyl-ACP reduction of the FAS-II cycle. ACP, Acyl Carrier Protein; KR, β-ketoacyl-ACP reductase activity; DH, β-hydroxyacyl-ACP dehydrogenase activity; ER, trans-2-enoyl-ACP reductase activity. Created with BioRender.com.

Among their multiple roles, fatty acids (FA) are structural components of cell membranes of almost all life forms. Even in archaea, with their lipids containing ether-linked isoprenoid chains, FA (Carballeira et al., 1997) and FA-containing lipids were described (Gattinger et al., 2002). Although the production cycle of these cell components is conserved, the enzymes that are responsible for the fatty acid synthesis (FAS) are arranged in different ways among species (Cronan, 2006). The FAS enzymes are essential for the survival of plants, animals, fungi, and bacteria, except for some Gram-positive bacteria (order Lactobacillales) that can bypass FAS inhibition by incorporating exogenous fatty acids into their phospholipids (Parsons et al., 2011; Yao and Rock, 2017). All these organisms share a similar enzyme machinery, with acetyl-coenzyme A (acetyl-CoA) as the initial substrate, while malonyl-CoA is used for chain extension (Brinster et al., 2009; Massengo-Tiassé and Cronan, 2009; Figure 1). There are two types of FAS systems in nature, type I and type II. The type I (FAS-I) is found in most eukaryotes aside plants, and in the *Corynebacteriales* order of Actinobacteria, composed mostly by mycolic acid-producing bacteria, such as mycobacteria and corynebacteria (Schweizer and Hofmann, 2004; Gago et al., 2011). Type II (FAS-II) system is found in mitochondria, plants, and in most bacteria, except for some of the aforementioned Corynebacteriales (in particular from the *Corynebacterium* genus) which rely exclusively on FAS-I megasynthase for FA synthesis (Gago et al., 2011; Marrakchi et al., 2014). The FAS-II is a dissociate system in which every enzymatic component is encoded by its own gene and catalyze one step of the route (White et al., 2005; Cronan, 2006). Additionally, mycobacteria make use of both FAS systems simultaneously. In this case, the systems work in tandem, with FAS-I producing FAs *de novo* with a bimodal product distribution (C_16–18_ and C_24-26_ in length; Gago et al., 2011), and FAS-II elongating them to C_18-30_ acyl chains (Marrakchi et al., 2014). In both FAS-I and FAS-II systems, after initiating with a condensing step involving acetyl-CoA and malonyl-CoA (FAS-I) or malonyl-ACP (FAS-II), FA chains are further extended by 2-carbon units in four sequential reactions during the elongation cycle: (1) condensation of acyl-ACP and malonyl groups—malonyl-CoA (FAS-I) or malonyl-ACP (FAS-II), (2) reduction of 3-ketoacyl-ACP, (3) dehydration of 3-hydroxyacyl-ACP, and (4) reduction of *trans*-2-enoyl-ACP to acyl-ACP (Figure 1). In FAS-II, the last step is performed by enoyl-ACP-reductases (ENRs).

**Figure 2:**
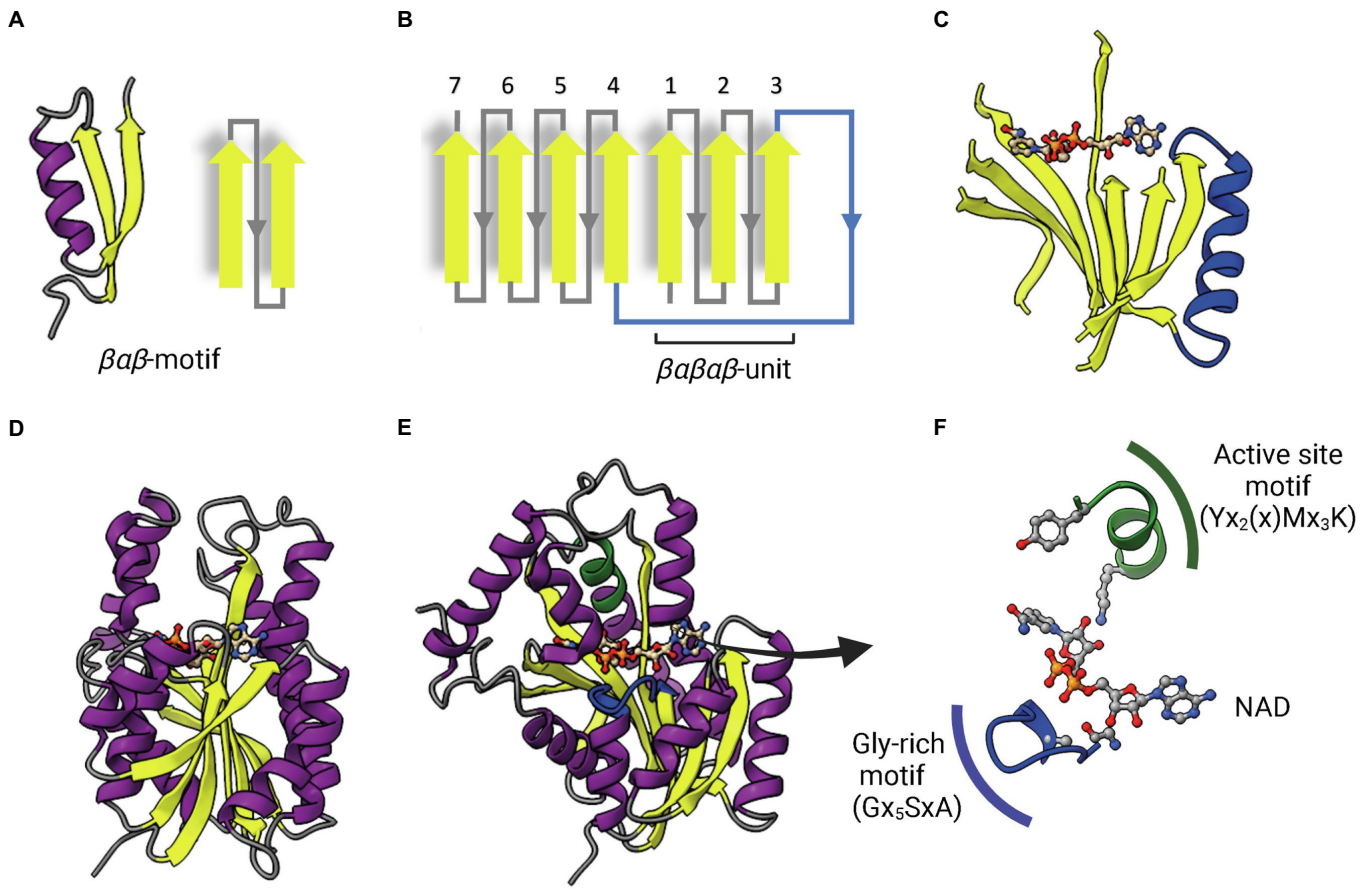
The dinucleotide-binding Rossmann fold. **(A)** A representative βαβ-motif and its topological representation. An α-helix (purple) connects two parallel β-strands (yellow). **(B)** Topological representation of the Rossmann fold. The βαβ-motifs form two sets of βαβαβ units that are connected by a long α-helix that function as a crossroad (in blue). **(C)** Representative parallel β-sheet of a Rossmann fold. The α-helix connecting the two sets of βαβαβ units through strands 3 and 4 is represented in blue. The NAD cofactor is also represented to indicate that the cofactor-binding site lies above this central parallel β-sheet. **(D)** Structure of InhA (PDB: 1ENY). The central parallel β-sheet is flanked by two sets of α-helices on each side. **(E)** InhA (PDB: 1ENY) with the central parallel β-sheet in lateral view. The active site motif Yx_2_(x)Mx_3_K and the cofactor-binding glycine-rich motif (Gx_5_SxA) are shown in green and blue, respectively. **(F)** Detailed view of NAD, the active site and Glycine-rich motifs. The NAD cofactor, the first glycine from the Gx_5_SxA motif (G14), catalytic tyrosine (Y158) and lysine (K165) from the signature motif Yx_2_(x)Mx_3_K are represented as Ball & Stick atomic models. Created with UCSF ChimeraX (Pettersen et al., 2021) and BioRender.com.

**Figure 3:**
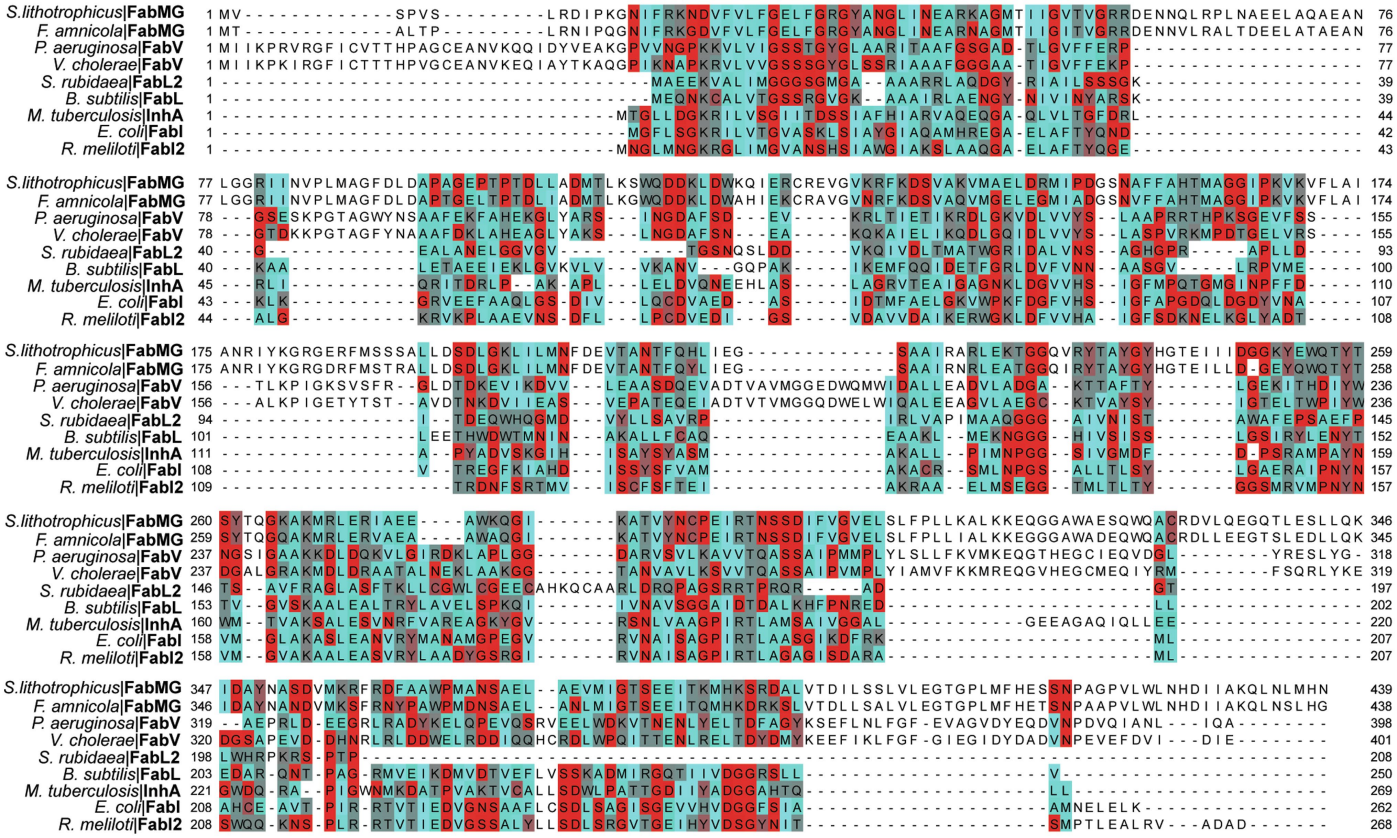
Multiple alignment of multiple ENRs from the SDR superfamily. Amino acids are colored based on their Turn Propensity, where darker shades of red correspond to a higher propensity. *E. coli*: *Escherichia coli*, *P. aeruginosa*: *Pseudomonas aeruginosa*, *B. subtilis*: *Bacillus subtilis*, *Serratia rubidaea*: *S. rubidaea*, *Rhizobium meliloti*: *R. meliloti*, *Vibrio cholerae*: *V. cholerae*, *S. lithotrophicus: Sideroxydans lithotrophicus, F. amnicola: Ferriphaselus amnicola*, *Mycobacterium tuberculosis*: *M. tuberculosis*. Each sequence was retrieved from the SwissProt database, except for the FabMG, which were retrieved from Kim et al. (2020). Multiple alignment was performed using Clustal Omega, and manually adjusted on JalView (Waterhouse et al., 2009).

ENRs are widely studied and considered great targets for drug discovery (White et al., 2005; Brinster et al., 2009; Massengo-Tiassé and Cronan, 2009). ENR enzymes use either NADPH or NADH as reduction agents to catalyze the reduction of *trans*-2-enoyl-ACP (Quémard et al., 1995; Massengo-Tiassé and Cronan, 2009). Most ENRs are short-chain dehydrogenase/reductase (SDR) superfamily members, including the FabI enzymes, which are related to *Escherichia coli* FabI, like the mycobacterial InhA. FabI enzymes are small (~30 kDa) proteins, widely distributed and constitute the most studied ENR type. Together with medium and long-chain dehydrogenases/reductases (MDR and LDR, respectively), SDR enzymes contain the dinucleotide-binding Rossmann fold, in which *βαβ*-motifs form two sets of *βαβαβ* units that make up a parallel β-sheet with 6 or 7 *β*-strands flanked in both sides by 3 or 4 *α*-helices (Figures 2A–D; Lesk, 1995; Kavanagh et al., 2008). The NAD cofactor binding site lies above the central parallel β-sheet (Figure 2C). The strand topology is also preserved: 7–6–5-4-1-2-3 (Figure 2B). Typically, there is a long crossroad connecting strands 3 and 4 that contributes to the binding site of the adenine ring of NAD (Figures 2C,D; Lesk, 1995). Differently from MDR and LDR enzymes, SDR members typically display a one-domain architecture (Figures 2D,E). According to cofactor and active site sequence motifs, SDR members are classified into five different SDR subfamilies: classical, extended, intermediate, divergent and complex (Kavanagh et al., 2008). Bacterial SDR ENRs are members of the divergent subfamily, containing the active site signature motif Yx_2_(x)Mx_3_K and the NAD binding motif Gx_5_SxA (Figure 2F). Interestingly, SDR members catalyze the transfer of the pro-*S* hydride from the dinucleotide cofactor, while MDRs catalyze the transfer of the pro-*R* hydride. The pro-*S* hydride transfer is favored by the way NAD binds in SDR members, in an extended conformation (Figure 2C; Kavanagh et al., 2008). Despite the structural and mechanistic similarities, these enzymes are considerably diverse in terms of primary structure, with identities ranging from 15 to 30% (Figure 3; White et al., 2005; Massengo-Tiassé and Cronan, 2009).

Furthermore, there is also non-SDR ENRs, such as the canonical FabK from *Streptococcus pneumoniae* and related proteins. FabK ENRs are flavin mononucleotide (FMN)-containing and NADH or NADPH-dependent enzymes from the flavin oxidoreductase family. Structurally, they are unrelated to the SDR ENRs, containing a conserved TIM barrel fold with eight α-helices and eight β-sheets alternated. This alternate ENR occurs in various bacterial species, such as *S. pneumoniae* and *Clostridioides difficile,* and have been considered a promising drug target. (Massengo-Tiassé and Cronan, 2009; Marreddy et al., 2019). We will now describe in more detail the FabI, FabV, FabL, FabK and novel bacterial ENR proteins found from soil metagenomes (FabL2, FabI2, FabMG), both in terms of structure and kinetic mechanisms.

### FabI

FabI is the major and most studied enoyl-ACP reductase enzyme of the FAS II system, which is found in several microorganisms, such as *Staphylococcus aureus*, *E. coli*, *Bacillus subtilis*, *Francisella tularensis*, *Burkholderia pseudomallei*, *Haemophilus influenza*, *Moraxella catarrhalis*, *Acinetobacter baumannii*, *Bacillus anthracis*, *Chlamydia trachomatis*, *Pseudomonas aeruginosa*, *Plasmodium falciparum*, and *Toxoplasma gondii*. Some bacterial species possess only FabI, while others contain an additional ENR from the FabL, FabV or FabK types (Khan et al., 2018). InhA (FabI homologue) is also present in *Mycobacterium tuberculosis* and other mycobacterial species (Banerjee et al., 1994; Quémard et al., 1995).

**Figure 4:**
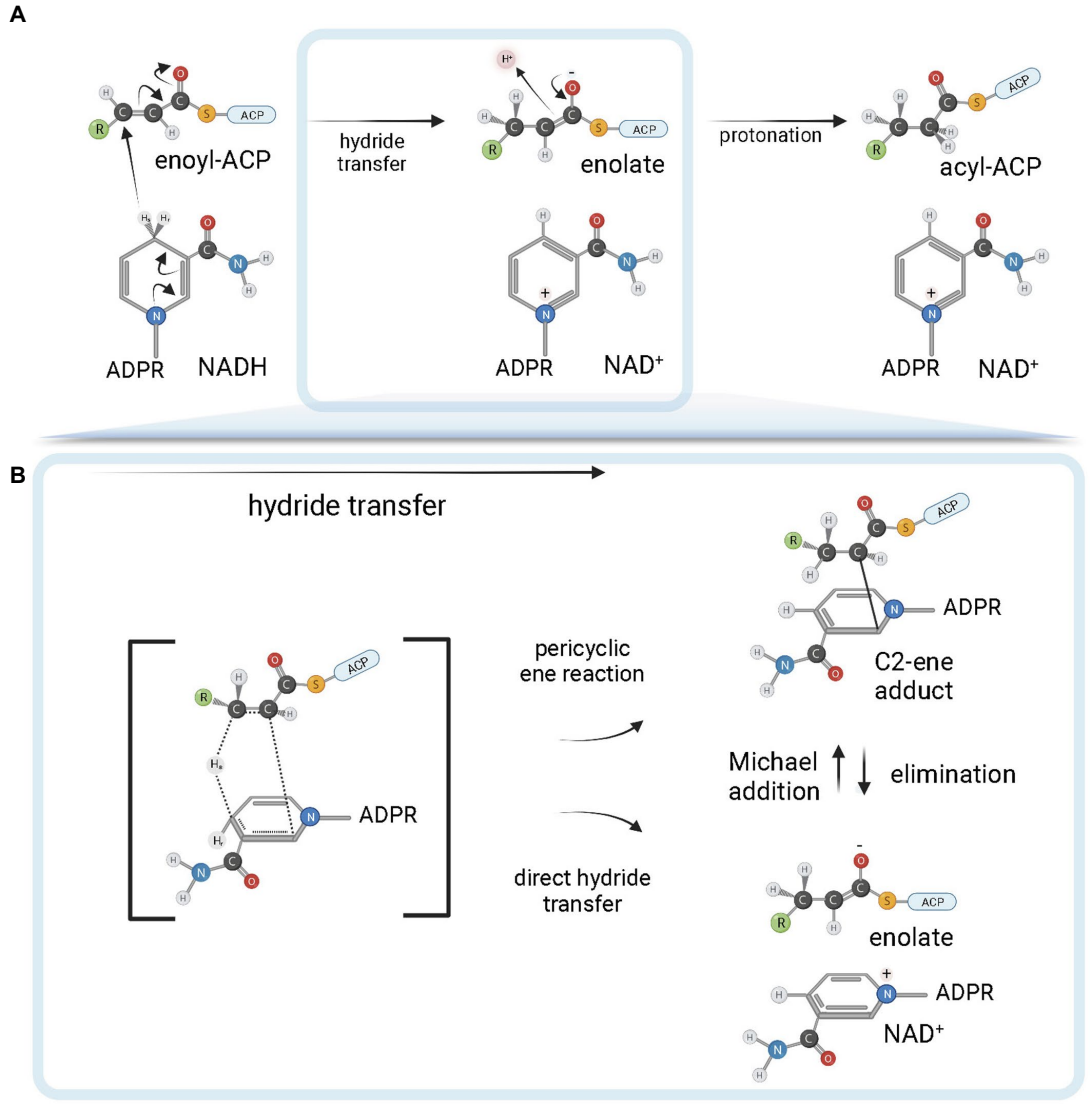
FabI reaction mechanism. **(A)** Reduction of trans-2-acyl-ACP (enoyl-ACP) to acyl-ACP by FabI enzymes. A reduced form of the dinucleotide cofactor (NADH or NADPH, depending on the enzyme) serves as the reductant species. In the first half-reaction, bacterial FabI enzymes catalyze the hydride transfer from the 4*S* hydrogen position of the nicotinamide ring (represented in the figure) of the cofactor to the C3 position (Cβ) of the α,β-unsaturated thioester of the enoyl-ACP substrate. An enolate intermediate is formed with the concomitant oxidation of NADH to NAD^+^. In the second half-reaction, the enolate intermediate is protonated, leading to the formation of the acyl-ACP product. **(B)** Covalent ene adduct intermediate between NADH and enoyl-ACP substrate. In this covalent intermediate species, there is a covalent bond between the C2 atom of the NADH nicotinamide ring and the C2 atom of the acyl substrate. Two alternate possibilities for hydride transfer are depicted. The hydride transfer could be the result of a pericyclic ene reaction from a transition state that leads to the C2-ene adduct or could be the result of a direct hydride transfer. In the latter, C2-ene adduct would be formed by Michael addition from the intermediate enolate and NAD^+^. Created with BioRender.com.

ENR FabI enzymes catalyze the reduction of *trans*-2-acyl-ACP substrates (enoyl-ACP) to acyl-ACP using either NADH or NADPH (depending on the enzyme) as reductant species (Figure 4; Massengo-Tiassé and Cronan, 2009). The chemical mechanism in bacterial FabI enzymes involves a nucleophilic conjugate addition of a hydride ion from the 4*S* hydrogen position of the nicotinamide ring of either NADH or NADPH to the C3 position (Cβ) of the α, β-unsaturated thioester of the enoyl-ACP substrate, followed by protonation at C2 (Cα) of the enzyme-stabilized enolate intermediate (Figure 4A; Saito et al., 1980; Quémard et al., 1995; Parikh et al., 1999). The conformation of the cofactor is modulated by the enzyme to facilitate the hydride transfer, with the 4*S* hydrogen pseudoaxially oriented relative to the nicotinamide ring (Bell et al., 2007). A breakthrough in the understanding of the reaction mechanism of enzymes catalyzing hydride transfers was made with the MDR superfamily member Ccr, a crotonyl-CoA reductase/carboxylase. It was shown that NADPH forms a covalent ene adduct intermediate with crotonyl-CoA (Rosenthal et al., 2014). Subsequently, a similar covalent adduct was found between NADH and an octenoyl-CoA substrate during the reaction cycle of InhA (Figure 4B), raising the interesting possibility that covalent adduct formation can be a conserved hydride transfer mechanism among dehydrogenases/reductases (Vögeli et al., 2018). This intermediate species has a covalent bond between the C2 of the NADH nicotinamide ring and the C2 atom of the acyl substrate. As it is already reduced at the C3 position, but not yet protonated at C2, this species is an intermediate between the two half-reactions of hydride transfer and protonation. Conceivably, the C2-ene adduct could be produced directly from an ene-shaped transition state by a pericyclic ene reaction or after hydride transfer by a Michael addition (Figure 4B). Apart from improving our understanding of the reaction mechanisms of FabI enzymes and dehydrogenases in general, the discovery of this covalent intermediate opens exciting new avenues for the development of novel high-affinity mechanistic inhibitors.

The protonation mechanism is still a matter of debate. Initially, it was suggested that the C1 carbonyl oxygen of the enolate anion intermediate could be protonated, with subsequent tautomerization leading to the reduced acyl product. The conserved tyrosine residue from the active site signature motif of *E. coli* FabI (Y156 in this enzyme—*Ec*FabI—and Y158 in *M. tuberculosis* InhA) was suggested to be the proton donor to the enolate anion, while the conserved lysine (K163 and K165, respectively) would stabilize the negative charge of the transition state (Baldock et al., 1998b). However, as subsequently found, the reduction reaction follows a specific stereochemical course, with hydride transfer at C3 and protonation at C2 occurring in the same face (*syn* addition), yielding (2*R*,3*S*)-[2,3-^2^H_2_]octanoyl-CoA from *trans*-2-octenoyl-CoA after ^2^H_2_ addition (Fillgrove and Anderson, 2000). As already observed, this defined stereochemical outcome is not compatible with a mechanism involving enol formation by protonation of the enolate oxygen (Schiebel et al., 2015), implicating a direct protonation of C2 (Cα carbon).

**Figure 5:**
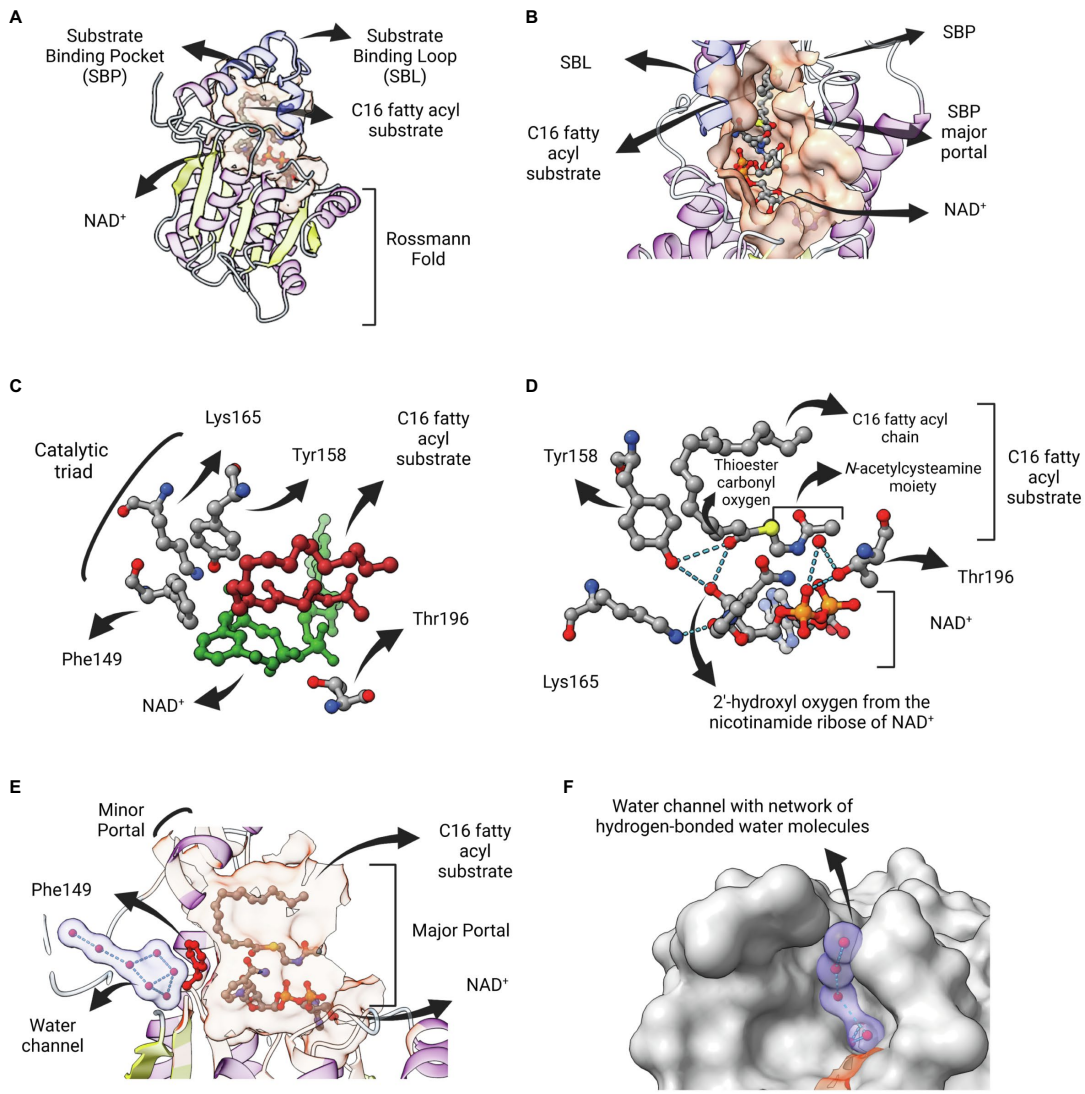
InhA bound to NAD and fatty acyl substrate. **(A)** Structure of the ternary complex of *Mycobacterium tuberculosis* InhA bound to NAD^+^ and a C16 fatty acyl substrate (PDB: 1BVR). Both cofactor-binding site and fatty acyl binding site lie within the same structural substrate binding pocket (SBP; beige surface). The substrate-binding loop (SBL) of mycobacterial FabIs like *M. tuberculosis* InhA (residues 196–219, in blue) are longer than their bacterial orthologues, presumably to accommodate longer fatty acyl substrates. The fatty acyl substrate can be viewed in its U-shaped conformation. The Rossmann fold is also represented. **(B)** View of the substrate binding pocket from the major portal. The U-shaped fatty acyl chain lies just ahead of the cofactor. **(C)** Detailed view of the catalytic triad (F149, Y158 and K165), together with T196, fatty acyl substrate and cofactor. **(D)** Hydrogen bond interactions among the catalytic Y158 and K165, the fatty acyl substrate and the NAD cofactor. **(E)** Lateral view of the substrate binding pocket of InhA displaying a network of water molecules inside a water channel. The residue F149 presumably gates the access of this water channel to the active site of InhA. This network of water molecules was proposed to be implicated in the protonation of the enolate intermediate. The minor portal is also indicated. **(F)** Surface view of InhA with the external part of the hydrogen-bonded network of water molecules inside the water channel. Binding pocket was identified using CASTp 3.0 (Tian et al., 2018). Created with UCSF ChimeraX (Pettersen et al., 2021) and BioRender.com.

The structural elucidation of a ternary complex containing InhA associated with both NAD^+^ and a C16 fatty acyl substrate (*trans*-2-hexadecenoyl-(N-acetylcysteamine)-thioester) gave new insights into the reaction mechanisms of ENR FabIs (Rozwarski et al., 1999). The fatty acyl substrate lies on the top of the cofactor and adopts a U-shaped conformation. Both fatty acyl and NAD^+^ binding sites lie within a pocket containing a major and a minor portal, with the substrate-binding loop (SBL) contributing with most of the hydrophobic residues that surrounds the fatty acyl chain (Figures 5A,B). The SBL was found to be longer in InhA from *M. tuberculosis* and mycobacterial orthologs than that of other ENR FabIs, comprising α-helix 6, a connecting loop, and part of α-helix 7 (residues 196–219). A longer binding crevice accommodates longer fatty acyl substrates, consistent with the fact that mycobacterial ENR FabIs, which are involved in mycolic acid biosynthesis, preferentially reduce long-chain substrates (>12 carbons).

Residue F149 is structurally analogous to S146 of the canonical SDR *E. coli* 7α-HSDH, a residue that is part of the catalytic triad of this enzyme (S146-Y159-K163). This led to the proposal that the catalytic triad in bacterial FabIs is F149-Y158-K165 (InhA positions), with F149 replaced by tyrosine for many FabIs (Figure 5C; Rozwarski et al., 1999). Interestingly, the only hydrogen bond between the fatty acyl substrate and the protein is between the hydroxyl oxygen from the conserved Y158 and the thioester carbonyl oxygen, with both of them making additional hydrogen bonds with the 2′-hydroxyl oxygen of the nicotinamide ribose of NAD^+^ (Figure 5D). The interaction between Y158 and the thioester carbonyl oxygen was unanticipated, since this residue adopts a different conformation in the structure of InhA bound to NADH elucidated previously (Dessen et al., 1995), referred to as the *out* conformation (Chollet et al., 2018). The ternary structure revealed that residue Y158 from InhA rotates upon fatty acyl substrate binding, orientating in a similar position adopted by the correspondent Y156 residue from *Ec*FabI (*in* conformation), and in this way being able to be hydrogen bonded with the thioester carbonyl oxygen. Kinetic isotope effect (KIE) studies of wild-type and mutant InhA (Y158F, Y158A, Y158S) corroborated this model, with Y158 functioning as an electrophilic catalyst, stabilizing the carbonyl enolate but not acting as a proton donor (Parikh et al., 1999). The ternary structure also revealed that the catalytic function of the conserved K165 residue is to hold the NADH in place and not to form an oxyanion hole for the thioester carbonyl oxygen, as previously suggested (Figure 5D; Baldock et al., 1998b). The role of K165 in cofactor binding was also confirmed in enzymatic studies of site-directed mutants (Parikh et al., 1999).

Residue T196 was also implicated in catalysis, by hydrogen bonding a water molecule that is also held by phosphate oxygen of NAD^+^ (Figure 5D). This structure also revealed the absence of any polar or ionizable residue close enough to C2 to serve as a proton donor, suggesting the proton could come from the solvent (Rozwarski et al., 1999). Aside its role modulating the conformation of NADH into the active site, contributing to the pseudoaxial positioning of the 4*S* hydrogen for hydride transfer (Bell et al., 2007), residue F149 would also serve as a barrier to a water channel and, upon rotation, would expose the active site to a network of hydrogen-bonded water molecules contained within this water channel that, ultimately, would lead to protonation (Figures 5E,F). This mechanistic model was further explored for *Sa*FabI, the ENR FabI from *S. aureus*. The catalytic efficiency of *Sa*FabI was compromised when residues stabilizing this conserved water wire were mutated. A pathway of proton transport was proposed, starting in a water chamber located inside the homo-tetrameric structure and followed by the network of water molecules inside the water channel (Schiebel et al., 2015). Moreover, the proposition that NAD(P)^+^ amide could be the source of the proton (Fillgrove and Anderson, 2000) was rejected because its hydrogen atoms are involved in a tight hydrogen bonding network (Schiebel et al., 2015).

Nevertheless, this picture for the protonation reaction is still controversial. In contrast to Parikh et al. (1999); Vögeli et al. (2018) found that Y158 is directed involved in protonation and is essential for the stereospecificity of the protonation reaction. The differences in data between the two studies were attributed to the method employed in the latter that directly measures KIE on protonation (Vögeli et al., 2018). The involvement of the intermediate covalent adduct, which was reported in this same study, was invoked to reconcile the *syn* addition of hydride transfer and protonation with a mechanism involving direct protonation from Y158. The (2S)-C2-ene adduct (Figure 4B) would lead to a change in hybridization of the C2 from sp2 to sp3, bringing the C2 closer to Y158 (Vögeli et al., 2018). A clear picture of the reaction mechanisms employed by ENR FabIs will require new mechanistic studies addressing these issues.

**Figure 6:**
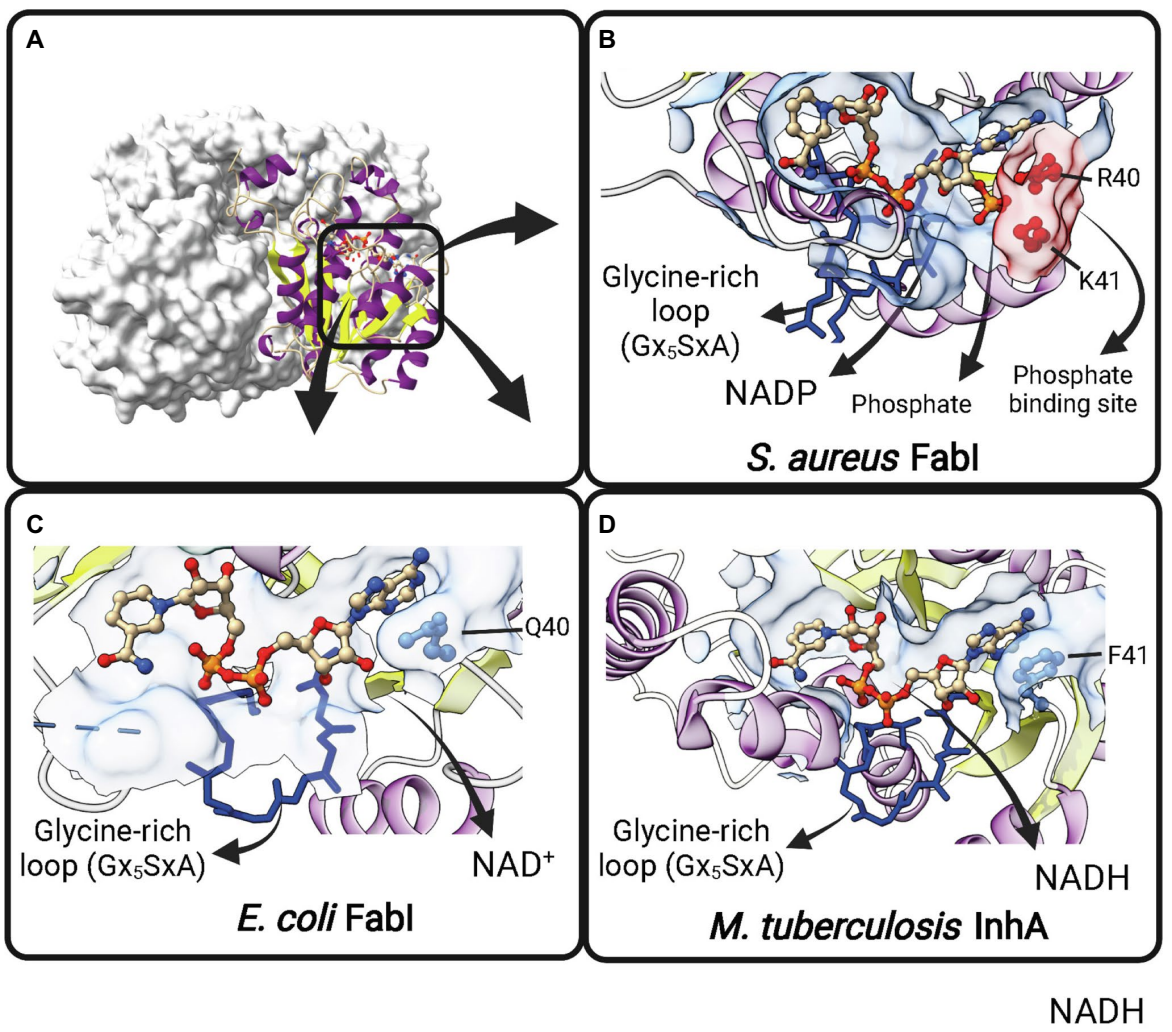
Cofactor-binding pockets of ENR FabIs. **(A)** Tetrameric structure of *E. coli* FabI (*Ec*FabI, PDB: 1DFI) as a prototypical tetrameric ENR FabI. Three protomers are represented in surface view and the fourth in cartoon representation. **(B)** Cofactor-binding site of *Staphylococcus aureus* FabI (*Sa*FabI) bound to NADP (PDB: 3GR6). The positively charged R40 and K41 residues (in red) that interact with the phosphate bound to the adenosine ribose of NADP are highlighted. **(C)** Cofactor-binding site of *Ec*FabI bound to NAD^+^ (PDB: 1DFI). The polar residue Q40 is found in the place of the positively charged residues required to accommodate the additional phosphate of NADP. **(D)** Cofactor-binding site of *Mycobacterium tuberculosis* InhA bound to NADH (PDB: 1ENY). The nonpolar F41 is found in the same approximate position as Q40 in *Ec*FabI and K41 and R40 in *Sa*FabI. **(B–D)** The Glycine-rich loop is highlighted in dark blue. Created with UCSF ChimeraX (Pettersen et al., 2021) and BioRender.com.

Contrary to early expectations (Weeks and Wakil, 1968), FabI is the single trans-2-enoyl-ACP reductase enzyme in *E. coli* (Bergler et al., 1996). It is encoded by *fabI* and utilizes substrates with different chain lengths (Campbell and Cronan, 2001). *E. coli* FabI is a NADH-dependent FabI. The lower NADPH-dependent activity previously reported for this enzyme (Bergler et al., 1996) could not be reproduced in later experiments (Heath et al., 2000b). *B. subtilis* and *Burkholderia. pseudomallei* FabI have also a marked preference for NADH over NADPH (Heath et al., 2000c; Liu et al., 2011). Moreover, *M. tuberculosis* InhA is strictly dependent on NADH (Quémard et al., 1995). However, *S. aureus* FabI (*Sa*FabI) have a clear preference for NADPH cofactor, despite being highly homologous to the others FabI enzymes (Heath et al., 2000b). The structural basis for this difference in cofactor specificity was found to be the presence of two positively charged residues (R40 and K41) lying close to the 2′-phosphate bound to the adenosine ribose of NADPH in *Sa*FabI (Xu et al., 2008; Priyadarshi et al., 2010; Figure 6). Residues at correspondent positions in NADH-dependent FabI enzymes from *E. coli* (Baldock et al., 1998b) and *M. tuberculosis* (Dessen et al., 1995) are polar (Q40 in *Ec*FabI) or nonpolar (F41 in *M. tuberculosis* InhA). Moreover, the importance of R40 and K41 for NADPH binding was directly evaluated by site-directed mutagenesis (Xu et al., 2008). Both R40Q and K41N mutants show at least 50-fold decrease in *k*_cat_/*K*_m_ for NADPH, with correspondent 5-7-fold increases in *k*_cat_/*K*_m_ for NADH (Xu et al., 2008).

Steady-state kinetics showed that the two substrates can bind to FabI *via* a sequential mechanism with few differences in the order of the substrate binding among microorganisms. InhA kinetic mechanism is not strictly ordered, but there is a preferred order of addition, with NADH binding first (Quémard et al., 1995). *B. pseudomallei* FabI-1 follows an ordered Bi-Bi kinetic mechanism in which NADH is the first substrate to bind in the active site (Liu et al., 2011), while a rapid equilibrium random kinetic mechanism has been proposed for *Haemophilus influenzae* FabI (Marcinkeviciene et al., 2001).

FabI has been shown to be essential for bacterial survival (Heath and Rock, 1995). The effect of FabI deficiency in a heat-sensitive FabI conditional mutant of *E. coli* impaired the completion of the elongation of long-chain saturated or unsaturated fatty acids without supplementation with exogenous FabI. The *fabI*(Ts) strains accumulate β-hydroxybutyryl-ACP at the nonpermissive temperature indicating that FabI is the only reductase required to complete both saturated and unsaturated fatty acid synthesis in the type II synthase system of *E. coli* (Heath and Rock, 1995). In addition, FabI seems to be an important control point in the fatty acid elongation cycle (Bergler et al., 1996). Due to the essential role that the fatty acids play in bacterial cell survival and the low degree of ENR sequence homology with mammalian targets, it has become one of the most attractive new antibiotic targets for the treatment of multidrug-resistant infections. FabI is well-studied and a validated target, in which several potent inhibitors with diverse chemical scaffolds have been discovered with potential antibacterial activity (Rana et al., 2020).

**Figure 7:**
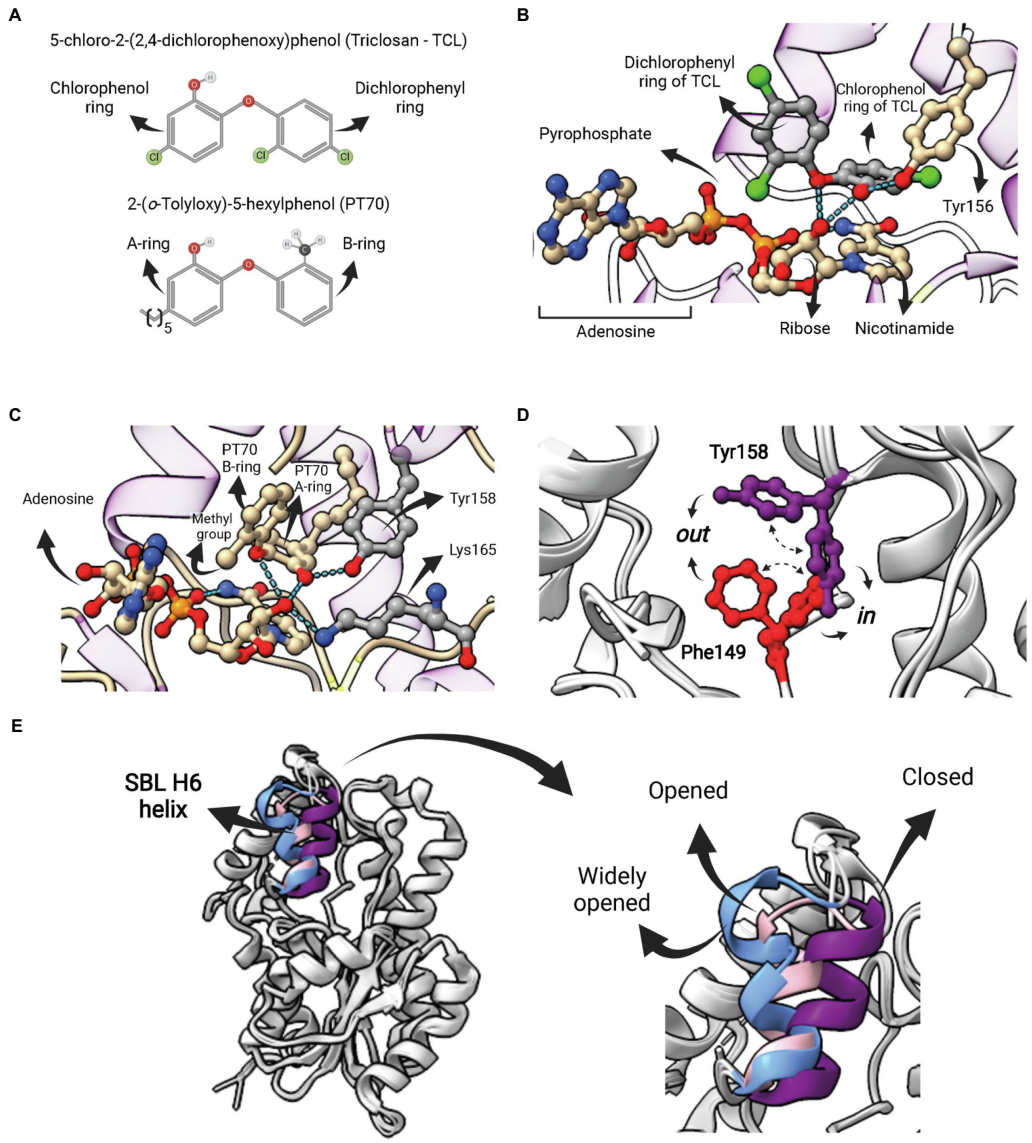
Competitive inhibitors of FabI—Triclosan and PT70. **(A)** Structure of 5-chloro-2-(2,4-dichlorophenoxy)phenol (Triclosan—TCL) and the TCL derivative 2-(o-Tolyloxy)-5-hexyphenol (PT70). **(B)** Ternary complex of *Ec*FabI bound to TCL and NAD (PDB:1QSG). Hydrogen bonds between the ether oxygen and the chlorophenol ring hydroxyl oxygen with the 2′-oxygen from the nicotinamide ribose of NAD are highlighted. **(C)** Ternary complex of InhA bound to NAD^+^ and PT70 (PDB: 2×23). Hydrogen bonds between the inhibitory compound and the catalytic Y158 and K165 residues are highlighted. This compound also makes hydrogen bonds with the 2′-nicotinamide ribose oxygen of NAD^+^. **(D)** Superposition of two InhA structures highlighting to possible conformations for F149 and Y158. In both cases, the *in* conformation is the one in which the residue points toward the active site, as opposed to the *out* conformation. In one structure (PDB: 4D0S) the F149 is in the *in* conformation, while Y158 is in *out* conformation. The second structure (PDB: 3FNH) the situation is the opposite (F149 *out*, Y158 *in*). Note that the configuration (F149 *in*, Y158 *in*) is not allowed due to steric hindrance. **(E)** Structural flexibility of the substrate-binding loop (SBL) helix 6 (residues 196–206) from InhA. This region is found in variable conformations in different structures, but they can be grouped in four types, each of them represented by one structure in the picture: disordered (4TRM), widely opened (1P44), open (2AQ8) and closed (3FNH). Potent slow-onset inhibitors usually induce the ordering of the SBL H6 helix into the closed conformation. The superposition of structures was performed using the Matchmaker tool from UCSF ChimeraX. Created with UCSF ChimeraX (Pettersen et al., 2021) and BioRender.com.

Current ENR FabI inhibitory compounds can be broadly considered as belonging to one of the following categories: (1) competitive inhibitors of the fatty acyl substrate, (2) molecules that form covalent adducts with the cofactor, and (3) bisubstrate inhibitors (Chollet et al., 2018). Different molecular structures that act as competitive inhibitors and bind into the fatty-acyl substrate-binding site were structurally characterized (Chollet et al., 2018; Rana et al., 2020). Among them, triclosan (TCL) and derivatives have been studied in more detail (Figure 7A). TCL, a trichlorinated biphenyl ether, is a common antibacterial additive used in consumer products and acts as a non-covalent FabI inhibitor (Heath et al., 1999). Triclosan is a slow, tight binding, reversible inhibitor of the *Ec*FabI, binding preferentially to the E-NAD^+^ form of the wild-type enzyme, with an inhibition constant value for this complex (*K*_1_) of 23 pM (Sivaraman et al., 2003). The crystal structure has shown that TCL forms a tightly associated ternary complex with the protein and the nicotinamide cofactor stabilized by hydrogen bonds and hydrophobic interactions (Figure 7B; Stewart et al., 1999). *Ec*FabI mutants (G93V, M159T and F203L) that confer resistance to triclosan showed a substantially reduced affinity binding to triclosan with *K*_1_ values of 0.2 μM, 4 nM, and 0.9 nM, respectively (Sivaraman et al., 2003). All the three FabI mutant residues are involved in the formation of the cofactor binding site, located close to the inhibitor (Qiu et al., 1999; Stewart et al., 1999). In addition, a mutation of Phe204 (equivalent to Phe203 in *Ec*FabI) to Cys in *S. aureus* that disrupts the formation of the FabI-NAD^+^-triclosan ternary complex confers resistance to triclosan in the *S. aureus* mutant (Fan et al., 2002). Triclosan also inhibits InhA from *M. tuberculosis* (Parikh et al., 2000) and *Mycobacterium smegmatis* (McMurry et al., 1999). Interestingly, triclosan is a weaker inhibitor of InhA compared to FabI. While triclosan binds preferentially to the E-NAD^+^ form of InhA, the affinity of triclosan for InhA is 10,000-fold lower than that for the *Ec*FabI (Parikh et al., 2000). On the other hand, *S. pneumoniae* (FabK), *B. subtilis* (FabI and FabL), and *P. aeruginosa* (FabI and FabV) are resistant to triclosan (Heath and Rock, 2000a; Heath et al., 2000b). Overexpression of the AcrAB-encoded multidrug efflux pump and MarA and SoxS, both positive regulators of *acrAB*, can also confer resistance to triclosan (Massengo-Tiassé and Cronan, 2009).

The poor inhibitory activity of TCL against InhA and its low antimicrobial activity against Mtb strains prompted efforts to discover more potent TCL derivatives. This led to the development of the most potent inhibitor of InhA currently available, the TCL derivative 2-(*o*-Tolyloxy)-5-hexyphenol (PT70; Figure 7A). This compound has an inhibition constant for the InhA-NAD^+^ complex of 22 pM (Luckner et al., 2010). As in the case of TCL, the ternary complex of PT70, NAD^+^ and InhA revealed that the compound occupies the same space of the fatty acyl substrate (Figure 7C; Luckner et al., 2010). The hydroxyl oxygen from the PT70 A-ring establishes hydrogen bounds with the catalytic Y158 and the 2′-nicotinamide ribose oxygen, which, in turn, also forms hydrogen bonds with the PT70 ether oxygen and with the catalytic K165 (Figure 7C). Tyrosine 158 is in the *in* conformation, pointing toward K165, in the same orientation found in the ternary complex with C16 fatty acyl substrate, opposed to the *out* conformation, found in all binary complexes of InhA with NAD^+^/NADH (Chollet et al., 2018). Residue F149, which together with Y158 and K165 makes up the catalytic triad of bacterial FabIs, is also found in two different conformations (Figure 7D). Interestingly, F149 *in* and Y158 *in* conformations are mutually exclusive, as they overlap in space (Figure 7D), and some inhibitors stabilize F149 *in* conformation, restricting Y158 into the catalytically inactive *out* conformation (Chollet et al., 2018).

Inhibition studies with TCL and TCL derivatives acting on ENR FabIs are interesting examples in which efforts were undertaken to optimize for compounds with longer residence times (*t_R_*) rather than necessarily for a lower equilibrium binding constant *K_d_*, an idea originally proposed by Copeland and colleagues (Copeland et al., 2006). Despite being a rapid reversible inhibitor for mycobacterial InhAs, TCL is a slow-onset inhibitor of various ENR FabIs (Xu et al., 2008; Lu et al., 2009; Schiebel et al., 2012; Chang et al., 2013). Moreover, TCL derivatives that are “slow-binders” of *M. tuberculosis* InhA were also developed, including the aforementioned PT70 (Luckner et al., 2010). The kinetic mechanism of these inhibitors was suggested to involve an induced-fit step, wherein the initial protein-ligand complex (EI) is followed by the ordering of the SBL H6 helix (196–206) into the *closed* conformation, which represents the EI* state (Li et al., 2014). Importantly, this segment is found in a wide range of conformations that can be described as disordered, widely open, open or closed (Figure 7E; Chollet et al., 2018). In the case of PT70, the rationale for its development was to reduce the ligand conformational flexibility to compensate for the entropic penalty associated with H6 helix ordering. The introduction of a methyl group *ortho* to the diphenyl ether linkage of the rapid reversible inhibitor 6PP was sufficient to generate a compound (PT70) with a residence time *t_R_* 14,000 times longer. In accordance with the proposed kinetic mechanism, the ternary complex of InhA, NAD^+^ and PT70 reveals that H6 helix is in the *closed* conformation (Luckner et al., 2010).

**Figure 8:**
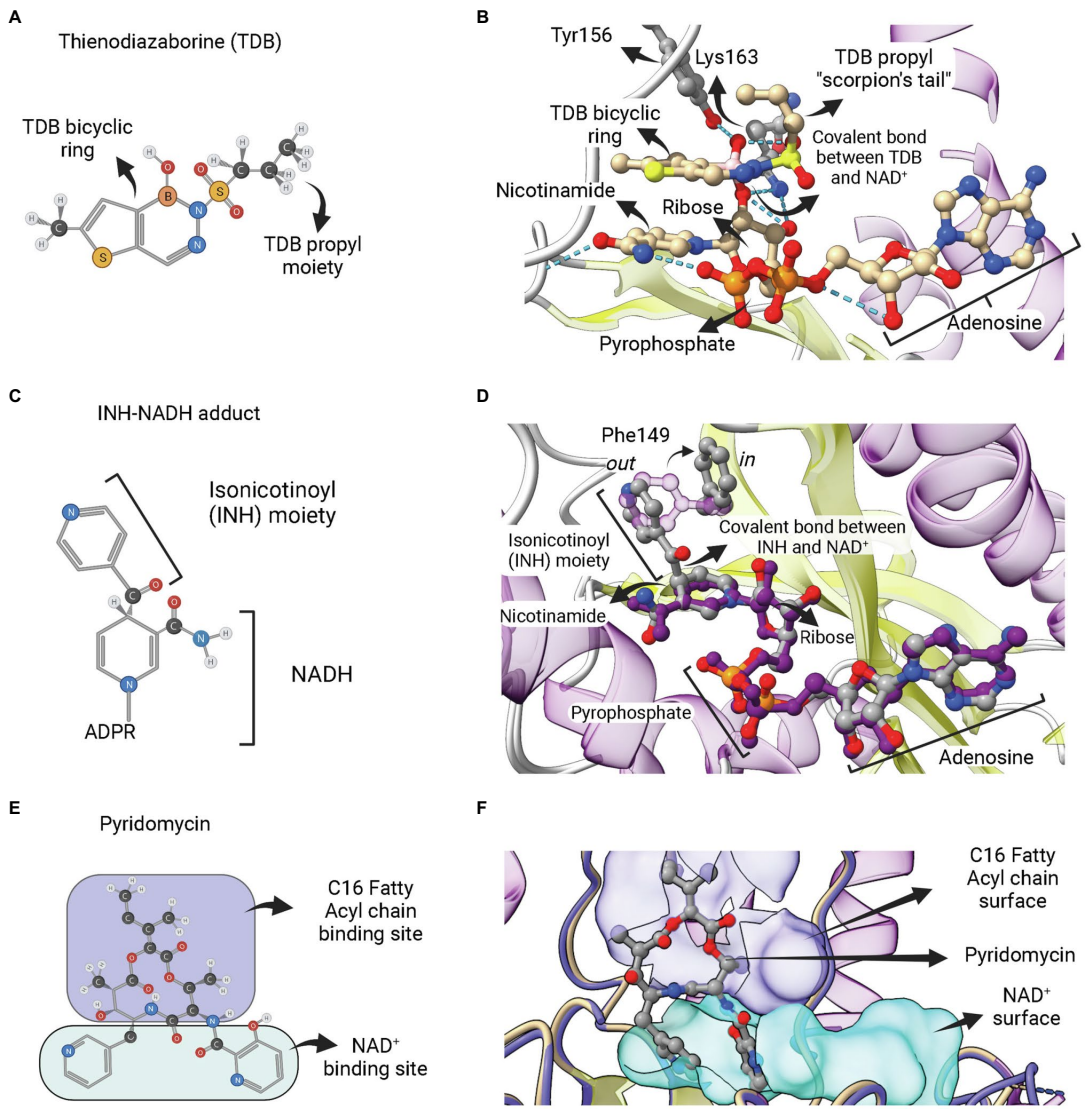
Adduct forming compounds and a bisubstrate inhibitor. **(A)** Structure of a diazaborine derivative (thienodiazaborine—TDB). **(B)** Ternary complex of *Ec*FabI bound to NAD^+^ and TDB (PDB: 1DFH). The TDB propyl moiety turns back in a conformation reminiscent to a scorpion’s tail (Baldock et al., 1996). The covalent bond between TDB and NAD^+^ is indicated. **(C)** Structure of the INH-NADH adduct. Only the nicotinamide portion of NADH is represented. **(D)** Comparison of the complex of InhA bound to the INH-NAD adduct (PDB: 1ZID) with the binary complex of InhA with NAD^+^ (PDB: 2AQ8). Both structures almost completely overlap, except for the conformation of F149, which rotates from the *out* conformation in the binary complex InhA-NAD^+^ to the *in* conformation in the enzyme complex with INH-NADH. This rotation is required to accommodate the isonicotinoyl (INH) moiety of the INH-NADH adduct. **(E)** Structure of the pyridomycin bisubstrate inhibitor. The portions that occupies the fatty acyl chain binding site and the NAD^+^ binding site are indicated. **(F)** Binary complex of InhA bound to pyridomycin (PDB: 4BII). The superposition of structures was performed using the Matchmaker tool from UCSF ChimeraX. Created with UCSF ChimeraX (Pettersen et al., 2021) and BioRender.com.

The second category of ENR FabI inhibitors includes adduct-forming compounds such as diazaborines (DAZ; Figures 8A,B) and the TB drugs isoniazid (INH; Figures 8C,D), ethionamide (ETH) and prothionamide (PTH). Diazaborines are a class of compounds that have a heterocyclic 1,2-diazine ring containing a boron atom (Baldock et al., 1998a). Several DAZ derivatives were synthesized in which the boron-containing diazine ring is fused to a five or six-membered ring. Five-membered thieno-fused derivatives (Figure 8A) are among the most potent antibacterial agents of this class, followed by the benzodiazaborines (Grassberger et al., 1984). Both thieno- and benzodiazaborines were found to inhibit ENR FabI by forming a covalent bond between the boron atom and the 2′-hydroxyl of NAD^+^ ribose. The diazaborine group binds in the enoyl substrate active site to form the diazaborine-NAD adduct (Figure 8B; Baldock et al., 1996). Diazaborine inhibitors also showed antibacterial activity against *M. tuberculosis* (Davis et al., 1998).

INH, a frontline antitubercular drug that targets InhA from *M. tuberculosis*, is a prodrug that requires activation by mycobacterial KatG catalase/peroxidase into an activated form of the drug (suspected to be an isonicotinic-acyl radical; Zhang et al., 1992, 1993; Heym et al., 1993; Johnsson and Schultz, 1994). Structural and mass spectrometry data revealed that the activated form of INH is found covalently linked to NADH within the active site of INH-inhibited InhA (Rozwarski et al., 1998). The INH-NAD adduct has an isonicotinic acyl group attached in the 4*S* position of the nicotinamide ring, replacing the hydrogen required for hydride transfer in the first half-reaction step of ENR FabIs (Figure 8C). INH-NAD adduct is a slow, tight binding competitive inhibitor for InhA (Rawat et al., 2003). The structure of InhA bound to INH-NAD adduct was found to be very similar to the binary InhA:NADH complex, with the main exception being the conformation of Phe149, which is rotated to accommodate the isonicotinic acyl group, going from the “out” to the “in” conformation (Figure 8D), according to the nomenclature proposed by Chollet et al. (2018). Rozwarski et al. (1998) also suggested that the INH-NAD adduct formation occurs within the enzyme’s active site, because they were unable to produce it without the addition of InhA. However, the formation of the INH-NAD adduct in the absence of InhA was achieved later on (Chollet et al., 2015). The preformed adduct was able to displace the cofactor NADH bound to InhA *in vitro* and only the reaction product with 4*S* configuration was found inside the structure of a binary complex between InhA and adduct (Chollet et al., 2015). Therefore, it is still unknown whether, in mycobacterial cells, INH-NAD adduct forms before binding to InhA or inside its active site.

Most INH resistance is related to mutations in the KatG and InhA enzymes. Mutation of InhA active site residues, such as Ser94 to Ala (S94A equivalent to G93V of *Ec*FabI) and Ile16 to Thr, decreases the enzyme affinity to NADH and INH, and strains with such mutations are resistant to INH. Structural studies of the InhA mutants reveal that INH resistance is directly associated with disruption of hydrogen bounds that stabilize NADH binding in the wild-type enzyme (Dessen et al., 1995).

ETH and PTH are thioamide analogues of INH that are used as second-line drugs to treat TB. They are structurally similar to INH, also require an activation step inside cells, and form NAD adducts that inhibit InhA (Wang et al., 2007). However, the activation mechanism was found to differ, with the monooxygenase EthA being required to activate ETH and PTH (Baulard et al., 2000; Wang et al., 2007).

The third category of ENR FabI inhibitors that act as bisubstrate inhibitors is represented by pyridomycin (Figure 8E). This compound is a natural product of the cyclodepsipeptide family, originally isolated as an antimycobacterial agent from *Streptomyces pyridomyceticus* (Maeda et al., 1953), which was later reclassified as *Dactylosporangium fulvum* (Shomura et al., 1986). InhA was identified as the target of this compound in *M. tuberculosis* after whole-genome sequencing of selected pyridomycin-resistant mutants (Hartkoorn et al., 2012). It was found as the first nonmimetic competitive inhibitor for the NADH-binding site of InhA (Hartkoorn et al., 2012). The structural elucidation of the binary complex containing pyridomycin bound to InhA revealed that the compound interacts with both the NADH- and lipid-binding sites (Figure 8F; Hartkoorn et al., 2014).

Several other classes of synthetic molecules, such as indoles, acrylamides, imidazoles, napthyridones, coumarins, pyrrolidine, pyridines, among others, have been reported as FabI inhibitors (in a nanomolar to micromolar range), which represent starting-point structures for the development of new antibacterial agents (Rana et al., 2020). A series of piperazine derivatives showed potent inhibition against InhA from *M. tuberculosis* (Rotta et al., 2015). Some natural products like cephalochromin (Zheng et al., 2007), verrulactone A and B (Kim et al., 2012b) are also capable of inhibiting the *Sa*FabI from *S. aureus*, with excellent *in vitro* antibacterial activity. The efforts devoted to the development of effective FabI-targeting antibiotics lead to the discovery of AFN-1252, CG400549 and FAB-1, currently in various stages of clinical development for the treatment MDR infections (Rana et al., 2020).

### FabV

**Figure 9:**
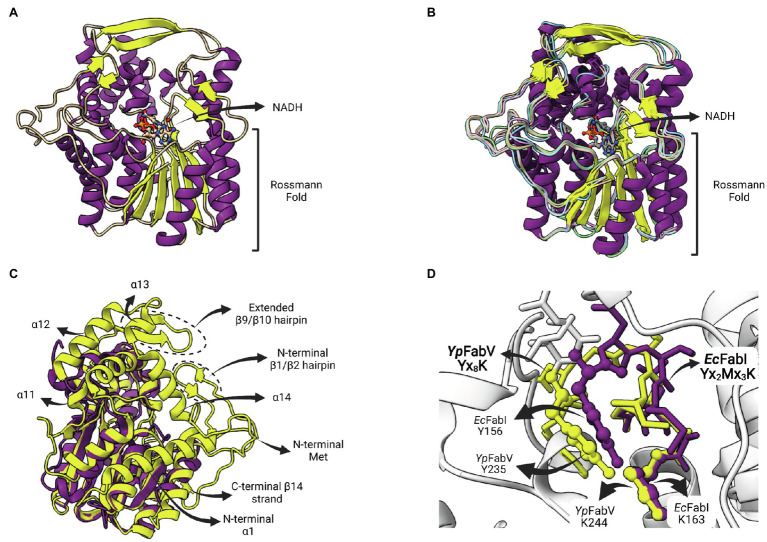
FabV structure. **(A)** Structure of *Yersinia pestis* FabV (*Yp*FabV—PDB: 3ZU3). The bound cofactor NADH and the eight-stranded Rossmann fold shared by FabVs are indicated. **(B)** Superposition of available FabV structures: *Y. pestis Yp*FabV (PDB: 3ZU3), *Xanthomonas oryzae Xo*FabV (PDB: 3S8M), *Burkholderia pseudomallei Bp*FabV (PDB:4BKO) and *Vibrio fischeri Vf*FabV (PDB:5XI0). **(C)** Superposition of *Yp*FabV (PDB: 3ZU3) with *Ec*FabI (PDB: 1C14). Structure of *Ec*FabI is depicted in purple while structure of *Yp*FabV is in yellow. Some structural elements of FabV ENRs not found in FabIs are indicated. **(D)** Structural comparison between the FabV extended active site signature motif Yx_8_K (from *Yp*FabV—PDB: 3ZU3) and the Yx_6_K (Yx_2_Mx_3_K) motif of FabIs (from *Ec*FabI—PDB: 1C14) reveals that the position of the catalytic lysine is almost identical, while the position of the catalytic tyrosine is very similar, in particular its hydroxyl oxygen atom. The superposition of structures was performed using the Matchmaker tool from UCSF ChimeraX. Created with UCSF ChimeraX (Pettersen et al., 2021) and BioRender.com.

The enzyme FabV is an enoyl-ACP reductase (ENR) originally identified in *Vibrio cholera* (*Vc*FabV), a Gram-negative bacterium that causes cholera in humans (Massengo-Tiassé and Cronan, 2008). Crystal structures from ENR FabV from *Xanthomonas oryzae* (Li et al., 2011), *Yersinia pestis* (Hirschbeck et al., 2012; Neckles et al., 2016), *B. pseudomallei* (PDB: 4BKO, not published) and *Vibrio fischeri* (Kwon et al., 2017) are available. ENR FabVs are 60% larger than other SDR members (except FabMG) but contain the typically conserved Rossmann fold, with an eight-stranded parallel β-sheet with three α-helices on one side and additional four helices on the other (Figure 9A; Hirschbeck et al., 2012). The structures available for the four FabVs are very similar (Figure 9B). However, when compared to other SDR ENRs, such as the *Ec*FabI, there are many regions exclusively found on FabVs (Figure 9C). In particular, there is a FabV-specific N-terminal region that includes a β-hairpin (β1/β2) and an extended α-helix (α1), more central structures, such as the extended β-hairpin (β9/β10) that lies at the top of the substrate-binding pocket and four helices that surround the active site (α11 to α14), and an additional C-terminal β-strand (β14; Hirschbeck et al., 2012). The catalytic tyrosine and lysine residues do not conform with the signature motif Yx_2_(x)Mx_3_K of the divergent subfamily of SDR ENRs (Kavanagh et al., 2008). In FabVs, they are spaced by eight residues (Yx_8_K), without the internal methionine. However, the two additional residues found in the signature motif of FabVs do not change the relative positions of the catalytic tyrosine and lysine, which lie in very similar positions relative to *E. coli Ec*FabI (Figure 9D).

FabV is expressed in other organisms than *V. cholera,* including important pathogens, such as *Burkholderia mallei*, *P. aeruginosa* and *Y. pestis* (Lu and Tonge, 2010; Zhu et al., 2010; Hirschbeck et al., 2012). Some organisms, such as *B. mallei* and *P. aeruginosa*, have both FabI and FabV, while *V. cholera* and *Y. pestis* contain only FabV (Rana et al., 2020). FabV catalyzes the reduction of the enoyl-ACP using NADH as the hydride donor. Kinetic studies have shown that the FabV from *B. mallei* (*Bm*FabV) features a sequential bi-bi catalysis mechanism, in which the NADH binding occurs first followed by the enoyl substrate (Lu and Tonge, 2010), like the mechanism reported previously for FabI (Fawcett et al., 2000; Marcinkeviciene et al., 2001). The Rossmann fold on the N-terminal part of FabV binds the nucleotide cofactor (Figure 9A) while the catalytic site that provides substrate specificity is located on the C-terminal part of the enzyme (Hirschbeck et al., 2012). Furthermore, FabV are found as monomers, differently from the others SDR ENRs FabI and FabL, that form tetrameric structures (Hirschbeck et al., 2012).

The deletion of *fabV* in *P. aeruginosa* results in a lower production of fatty acids and slower growth of these strains. Furthermore, mutation of *fabI* has no effect on growth or the fatty acid profiles, suggesting that FabV is the main ENR of this organism (Huang et al., 2016). Considering the successful use of FabI as a drug target, the development of FabV inhibitors in drug discovery efforts is underway. Triclosan is a potent inhibitor of FabI from different organisms, but its inhibition of FabV is weak and reversible (Massengo-Tiassé and Cronan, 2008; Lu and Tonge, 2010). Accordingly, it was shown that *P. aeruginosa* is resistant to TCL due to the presence of FabV, since the deletion of the *fabV* gene causes sensitivity to this compound (Zhu et al., 2010; Huang et al., 2016).

Inhibitory studies with compounds containing the 2-pyridone scaffold revealed that they behave as competitive inhibitors against FabV from *Y. pestis* (*Yp*FabV), with Ki values of 1–2 μM, being considerably more potent than TCL (Ki value of 71 μM; Hirschbeck et al., 2012). Inhibitory studies with the *Yp*FabV T276S mutant have shown that the activities of different classes of inhibitors are affected when compared to the wild-type enzyme. The affinity is lower for diphenyl ether inhibitors, while it is higher for 2-pyridone inhibitors and do not change for 4-pyridone compounds (Neckles et al., 2016). The T276 residue is located at the SBL on the N-terminus, and mutations in this residue change the size of the active site. This study was important to elucidate the importance of residue T276 for the activity of FabV, providing a framework for the development of novel inhibitors based on structure–activity relationships for this enzyme (Neckles et al., 2016).

### FabL

**Figure 10:**
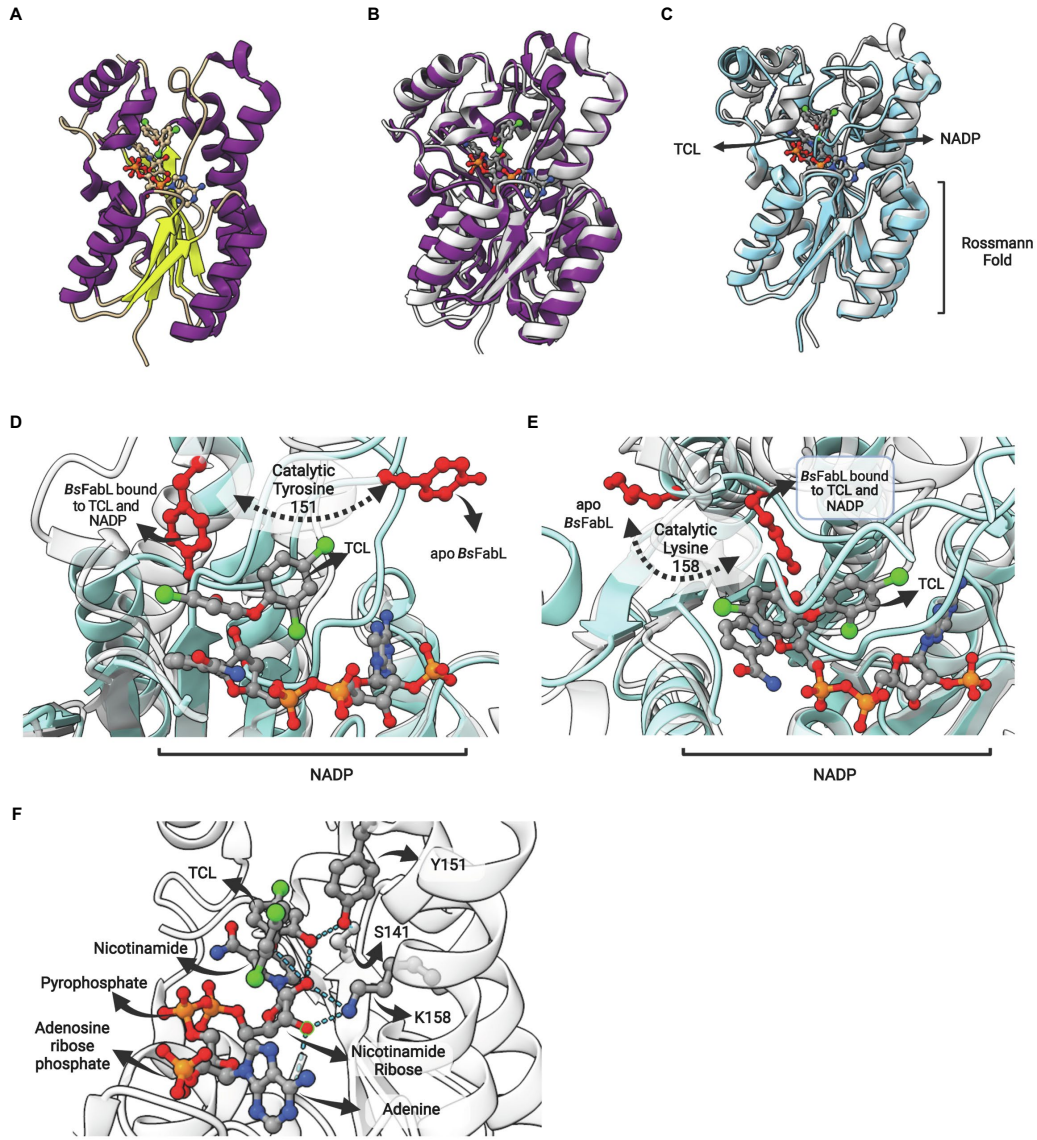
FabL structure: general view and interaction with triclosan. **(A)** Structure of *Bacillus subtilis* FabL (*Bs*FabL—PDB: 3OID) bound with NADP. **(B)** Superposition of *Ec*FabI (PDB: 1C14) with *Bs*FabL (PDB: 3OID). **(C)** Superposition of the ternary complex of *Bs*FabL bound to NADP and triclosan (TCL; PDB: 3OID) with the apo *Bs*FabL (PDB: 3OIC). **(D)** and **(E)** The position of both catalytic tyrosine and lysine reveals extensive conformational changes in the active site upon complex formation. **(D)** Position of the catalytic tyrosine (Y151 in *Bs*FabL, in red) in the apo enzyme (PDB: 3OIC) and bound to TCL and NADP (PDB: 3OID). **(E)** Position of the catalytic lysine (K158 in *Bs*FabL, in red) in the apo enzyme (PDB: 3OIC) and bound to TCL and NADP (PDB: 3OID). **(F)** Hydrogen bonds between NADP, TCL and the catalytic residues (Y151 and K158, PDB: 3OID). The superposition of structures was performed using the Matchmaker tool from UCSF ChimeraX. Created with UCSF ChimeraX (Pettersen et al., 2021) and BioRender.com.

FabL (or YgaA or enoyl-ACP reductase III) is an ENR encoded by the *ygaA* gene that was first described by Heath et al. (2000c) in *B. subtilis* (*Bs*FabL). This enzyme is a tetramer comprised of protomers containing 250 amino-acid residues in length (subunit MW = 27,178 Da) and has a strict requirement for NADPH as cofactor (Heath et al., 2000c). The crystallographic structure reveals that *Bs*FabL has the classical Rossmann fold (Figure 10A) and, albeit only 25% identical in sequence with *Ec*FabI, they are very similar in structure (Figure 10B; Kim et al., 2011). FabL enzymes have a signature motif with the expected spacing for the divergent subfamily of SDR ENRs (Yx_6_K), but without the internal methionine (Yx_2_(x)Mx_3_K). The extended signature *Ser-Xn-Tyr-Xn-Tyr-X6-Lys*, including the highly conserved Ser and Tyr residues involved in a hydrogen bond network in FabIs (White et al., 2005), is replaced by *Asn-Xn-Ser-Xn-Tyr-X6-Lys* in the *Bs*FabL, as is also found for FabG and other SDR members. The positions of the functional groups suggest that the active site of FabL is a hybrid between that of FabI and FabG (Kim et al., 2011).

The enzyme *Bs*FabL is inhibited by triclosan (TCL), presenting an IC50 for this compound of 16 μM. This value is eight times higher than what is found for *Ec*FabI (2 μM), but identical to the IC50 of TCL for *B.subtilis Bs*FabI (Heath et al., 2000c). However, TCL is a slow-onset inhibitor for *Bs*FabI, forming a stable NAD–triclosan ternary complex in a time-dependent manner, while for *Bs*FabL it is a reversible inhibitor (Heath et al., 2000c). In fact, FabL is responsible for triclosan resistance in *B. subtilis* and the inclusion of the *fabL* gene in high-copy-number plasmids leads to increased triclosan tolerance in *E. coli* (Heath et al., 2000c). Noteworthy, FabL is being used as a triclosan selection marker in *B. subtilis/E. coli* shuttle vectors (Mordukhova and Pan, 2020).

The comparison of the apo form with *Bs*FabL bound to TCL and NADP revealed that some structural regions surrounding the lipid-binding site are disordered in the apo form, and, upon NADH and TCL binding, these regions are drastically reoriented (Figure 10C). The catalytic tyrosine (Y151) and lysine (K158) of the signature motif are both completely reoriented upon NADP and TCL binding, positioning these residues for cofactor and fatty acyl binding (Figures 10D,E). Indeed, the analysis of ligand binding interactions reveals the pivotal role of Y151 and K158 in cofactor binding and in the interaction with TCL, positioned into the fatty acyl binding site (Figure 10F). The hydroxyl oxygen from Y151 hydrogen bonds with both S141 and the hydroxyl oxygen from the chlorophenol ring of TCL. On the other hand, K158 hydrogen bonds with both 2′- and 3′-hydroxyl oxygens from the nicotinamide ribose, as expected from its known role in cofactor stabilization in other SDR enzymes. Direct TCL-NADP interactions were also detected, with 2′-hydroxyl oxygen forming hydrogen bonds with both the hydroxyl oxygen of the TCL chlorophenol ring and ether oxygen (Figure 10F).

### Novel FabIs From Soil Metagenomes (FabL2, FabI2, FabMG)

To explore the possibility that the extensive and long-term use of TCL have led to the emergency of TCL-resistant bacteria in the environment, and that the soil could represent an environmental source of antibiotic resistant genes for human pathogenic bacteria, Khan et al. (2016) investigated the TCL resistome using a functional metagenomics-based screening. Samples from alluvial soil and industrially contaminated soil were used for metagenomic library construction. TCL-resistant clones were selected, subcloned, and the gene responsible for TCL resistance identified. Aside the identification of homologues of prototypic FabI, FabV, FabK or FabL ENRs, novel candidate ENR sequences were identified, presenting similarity with 7-α hydroxysteroid dehydrogenase (7-AHSDH), FabG or containing an YX_7_K signature motif (Khan et al., 2016).

The 7-AHSDH-like protein was characterized in more detail in another study and referred to as FabL2. This sequence presented significant homology with sequences annotated as 7-AHSDH in Epsilonproteobacteria (69 to 96%), followed by FabL (41%). Its coding sequence functionally complemented the *fabI*(Ts) *E. coli* mutant JPP1111 strain and conferred complete TCL tolerance. Interestingly, FabL2-like sequences were found among pathogenic Epsilonproteobacteria, in many cases along with FabI ENRs (Khan et al., 2018). The recombinant enzyme possesses NADPH-dependent ENR activity, but, albeit the higher similarity, did not exhibit 7-AHSDH activity. The modeled structure has a typical Rossmann fold containing a 7-stranded parallel β-sheet between two sets of three α-helices. The structural comparison between FabL and FabL2 revealed the presence of an extra 6-residue long loop in the latter (96–101). Based on molecular dynamics (MD) simulations, it was proposed that this loop might be involved in TCL resistance. Effectively, removal of the extra loop by site-directed mutagenesis resulted in loss of TCL resistance (Khan et al., 2018).

Another alternate SDR ENR obtained from the same metagenome screening (Khan et al., 2016) was characterized in more detail (Khan et al., 2019). This sequence is similar to FabI sequences (30%) but contains the Yx_7_K signature motif; hence, the name FabI2. FabI2 complemented ENR activity and *in vitro* is a NADH-dependent ENR. Additionally, a model of FabL2 revealed a Rossmann fold with a 7-stranded parallel β-sheet flanked by three α-helices on each side.

**Figure 11:**
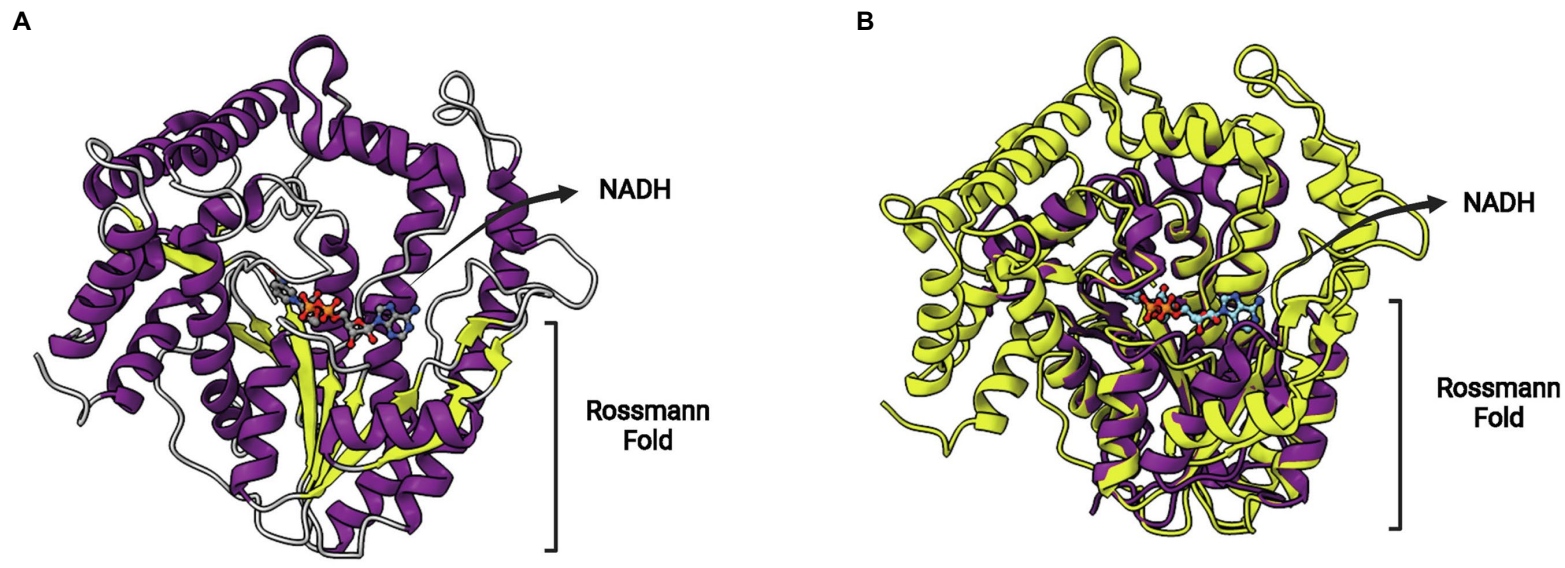
FabMG structure. **(A)** Structure of FabMG identified from a TCL-resistant clone in a soil metagenomics study (PDB: 6KIA). A bound NADH cofactor and the Rossmann fold are indicated. **(B)** Superposition of FabMG (represented in yellow, PDB: 6KIA) with *Ec*FabI (in purple, PDB: 1C14). The superposition of structures was performed using the Matchmaker tool from UCSF ChimeraX. Created with UCSF ChimeraX (Pettersen et al., 2021) and BioRender.com.

A novel hypothetical protein conferring resistance to TCL (protein AH4-3), obtained in the same functional screen, was also studied in more detail. Differently to the previous sequences described above (FabL2 and FabI2), this protein does not show any sequence similarity to proteins of known function (Kim et al., 2020). However, crystal structures were obtained and revealed that this protein is a novel SDR-type ENR protein. As is the case for FabV members, FabMG proteins are considerably larger than typical SDR proteins. The metagenome-derived FabMG has 438 residues, with a core Rossmann fold structure from which long α-helices project upwards (Figure 11A). The superposition of FabMG with *Ec*FabI reveals the conserved Rossmann fold above which the cofactor lies and a fatty acyl-binding site with extensive differences (Figure 11B). This ENR displays a signature motif with a shorter spacer, Yx_5_K. The ENR activity of FabMG was also confirmed in complementation studies using the *fabI*(Ts) *E. coli* mutant JPP1111 strain. Moreover, site-directed mutants Y260F and K266A were unable to complement this same strain, confirming the functional importance of Yx_5_K for its ENR activity.

### FabK

Although members of the SDR superfamily make up the most common type of ENRs in bacteria, a non-SDR ENR is found in some organisms, like *S. pneumoniae, C. difficile* and other Gram-positive bacteria (Marrakchi et al., 2002; Massengo-Tiassé and Cronan, 2009). FabK, a non-SDR ENR protein, was first described in *S. pneumoniae* (Heath and Rock, 2000a). This bacterium has a complete set of *fab* genes, except for *fabI.* FabK was found as an open reading frame (ORF) encoding a 34 KDa protein, demonstrated to have activity in an enoyl-ACP reductase coupled-assay system and to be resistant to triclosan inhibition (Heath and Rock, 2000a). FabK has been identified in other organisms, such as in *Streptococcus mutans*, *Thermotoga maritima, C. difficile, Porphyromonas gingivalis* and *Enterococcus faecalis,* the latter having both FabI and FabK enzymes, and has been studied as a drug target (Hoang and Schweizer, 1999; Heath and Rock, 2000a; Zhu et al., 2010; Nakashima et al., 2011; Khan et al., 2018).

**Figure 12:**
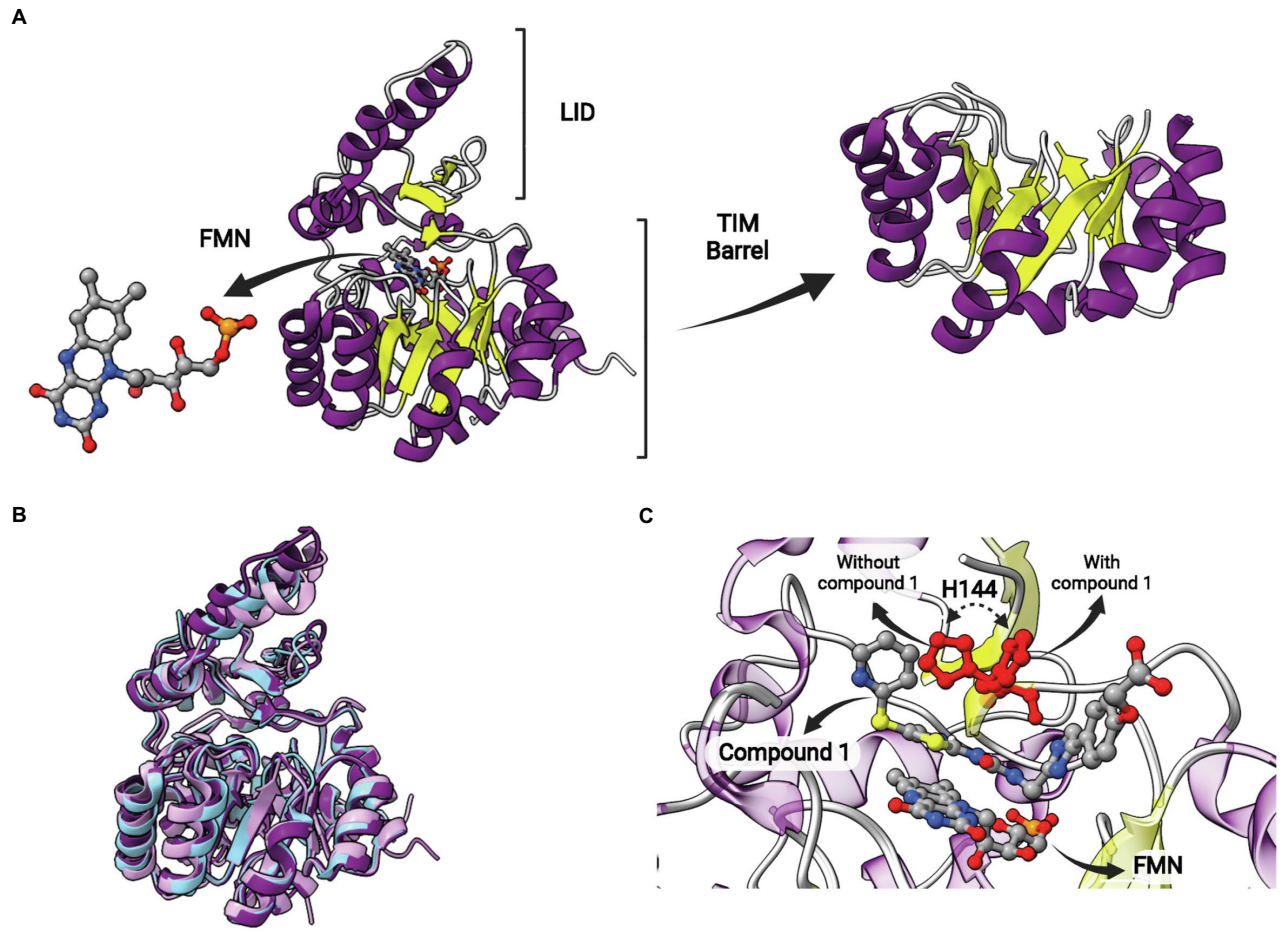
FabK structure. **(A)** Structure of *Streptococcus pneumoniae* FabK (*Sp*FabK, PDB: 2Z6I). The LID and the TIM barrel fold are indicated. The latter, together with a bound flavin mononucleotide (FMN) cofactor, are represented in more detail. **(B)** Superposition of *Porphyromonas gingivalis* FabK (*Pg*FabK, PDB: 4IQL, represented in pink) with *Thermotoga maritima* FabK (*Tm*FabK, PDB: 5GVH, represented in cyan) and *S. pneumoniae* FabK (*Sp*FabK, PDB: 2Z6I, represented in purple). **(C)** Superposition of *Sp*FabK bound to FMN cofactor (PDB: 2Z6I) with the ternary structure of *Sp*FabK bound to FMN and a phenylimidazole derivative inhibitor (compound 1; PDB: 2Z6J). The orientation of a putative catalytic histidine residue (H144) rotates upon inhibitor binding. The superposition of structures was performed using the Matchmaker tool from UCSF ChimeraX. Created with UCSF ChimeraX (Pettersen et al., 2021) and BioRender.com.

This protein is structurally different from other ENRs, displaying a TIM barrel fold with eight α-helices and eight β-sheets alternated (Figure 12A). It is a flavin mononucleotide (FMN)-containing enzyme that belongs to the NAD(P)H-dependent flavin oxidoreductase family (Massengo-Tiassé and Cronan, 2009). *S. pneumoniae* FabK (*Sp*FabK) was the first to have its crystal structure elucidated (Saito et al., 2008). FabK forms homodimers, and the protomers are highly similar to each other, with low mean square deviation (*rmsd*) values. Some differences between FabK and SDR ENR are visible, such as the α-helical insertion domain near C terminus, that is not present in FabK (Saito et al., 2008). FabK shows a two-step ping-pong catalytic mechanism that couples the reduction of a flavin mononucleotide (FMN) to the oxidation of the NADH. The NADH acts indirectly reducing the flavin cofactor which will reduce the double bond of the enoyl-ACP subtract (Marrakchi et al., 2003; Saito et al., 2008; Massengo-Tiassé and Cronan, 2009; Kim et al., 2012). The structures of bacterial FabK from *P. gingivalis* (*Pg*FabK) and *T. maritima* (*Tm*FabK) were also elucidated. They share a very similar structure (Figure 12B), but some differences among FabK proteins can be found (Saito et al., 2008; Kim et al., 2012; Ha et al., 2017; Hevener et al., 2018). *Pg*FabK show selectivity for NADPH over NADH, and it occurs because the 2′-ribosyl phosphate group of NADPH make electrostatic interactions with Lys263 and His46, while NADH binds the *Sp*FabK at corresponding residues Ala267 and Pro47. *Tm*FabK shows the same NADH preference as the *S. pneumoniae* enzyme (Saito et al., 2008; Ha et al., 2017; Hevener et al., 2018). The residues among three species (*S. pneumoniae, P. gingivalis* and *T. maritima*) are 34% identical and the active site similarity is significantly higher, with most of the differences found for *Pg*FabK. The FMN-binding region from Ala188 to Phe195 is conserved among all the species, except for a methionine substitution in *Tm*FabK at the position 191 (Saito et al., 2008; Hevener et al., 2018).

First described as a triclosan-resistant protein, organisms that present FabK are typically more resistant to TCL than the organisms that present FabI, being the *fabK* gene considered one TCL-resistant determinant mechanism, as well as *fabV, fabL* and *acrB*, an efflux pump (Heath and Rock, 2000a; Khan et al., 2018). The expression of FabK in *E. coli* can rise the inhibitory concentration of TCL by 4 times, evidencing the ability of FabK to confer resistance to TCL (Marrakchi et al., 2003; Massengo-Tiassé and Cronan, 2009). Although *E. faecalis* has both FabI and FabK, none of them mediate TCL resistance, being the *fabK* knockout viable without fatty acid supplementation and having no difference in the inherent TCL resistance (Zhu et al., 2010).

Some naturally occurring compounds were found to efficiently inhibit FabK, as Atromentin and Leucomelone, the first specific *Sp*FabK inhibitors. Atromentin and Leucomelone inhibited this enzyme with IC_50_ values of 0.24 and 1.57 μM, respectively, while not inhibiting the FabI of either *E. coli* or *S. aureus* (Zheng et al., 2006; Massengo-Tiassé and Cronan, 2009). Leucomelone showed the ability to inhibit *Sp*FabK with Ki and Km values of 4.1×10^−7^ M and 1.9×10^−4^ M, respectively. However, neither of the compounds displayed antibacterial activity, possibly due to the activities of efflux pumps (Zheng et al., 2006). In addition to natural compounds, AG205 was described as an efficient FabK inhibitor, showing IC_50_ of 1.5 μM, exhibiting antibacterial activity of 1–8 μg/ml against most of the tested *S. pneumoniae* isolates while no inhibition was observed against bacteria possessing FabI. This antibacterial activity was found to be bacteriostatic rather than bactericidal (Takahata et al., 2006).

Phenylimidazoles derivatives have also been studied as FabK inhibitors. These compounds inhibit recombinant FabK and are potent antibacterial agents against *S. pneumoniae*. Some of them are specific inhibitors for FabK, while others were found to be dual inhibitors, acting on both FabK and FabI (Kitagawa et al., 2007a,b). The most active specific-FabK phenylimidazole revealed an IC_50_ value of 0.14 μM and a MIC value against *S. pneumoniae* of 0.5 μg/ml. On the other hand, the most active 4-pyridone derivative compound showed IC_50_ of 0.38 ± 0.07 μM and 0.0045 ± 0.0007 μM for *Ec*FabI and *Sp*FabK, respectively. The MIC against *S. aureus* and *S. pneumoniae* of this same compound was >32 and 0.5 μg/ml, respectively (Kitagawa et al., 2007a,b). Phenylimidazoles also showed activity against a clinically dominant ribotype 027 *C. difficile*, with MIC values of 8 and 2 μM in the absence and presence of palmitic acid or fatty acid mix, respectively. The enzymatic inhibition of the same compound for *C. difficile* FabK was demonstrated with IC_50_ of 3.31 ± 0.046 μM (Marreddy et al., 2019). The structure of *Sp*FabK was elucidated in the presence of a phenylimidazole derivative (compound 1; Figure 12C; Saito et al., 2008). A conserved histidine (H144), suggested to play a role in catalysis as a proton donor/acceptor, is found to rotate upon complex formation with compound 1 (Figure 12C). As observed for the catalytic tyrosine in SDR ENRs, this rotation could be relevant in catalysis to position histidine toward the reactants, but the catalytic mechanism of FabKs was not studied in more detail yet.

### Evolution and Diversity of Enoyl-ACP Reductases

**Figure 13:**
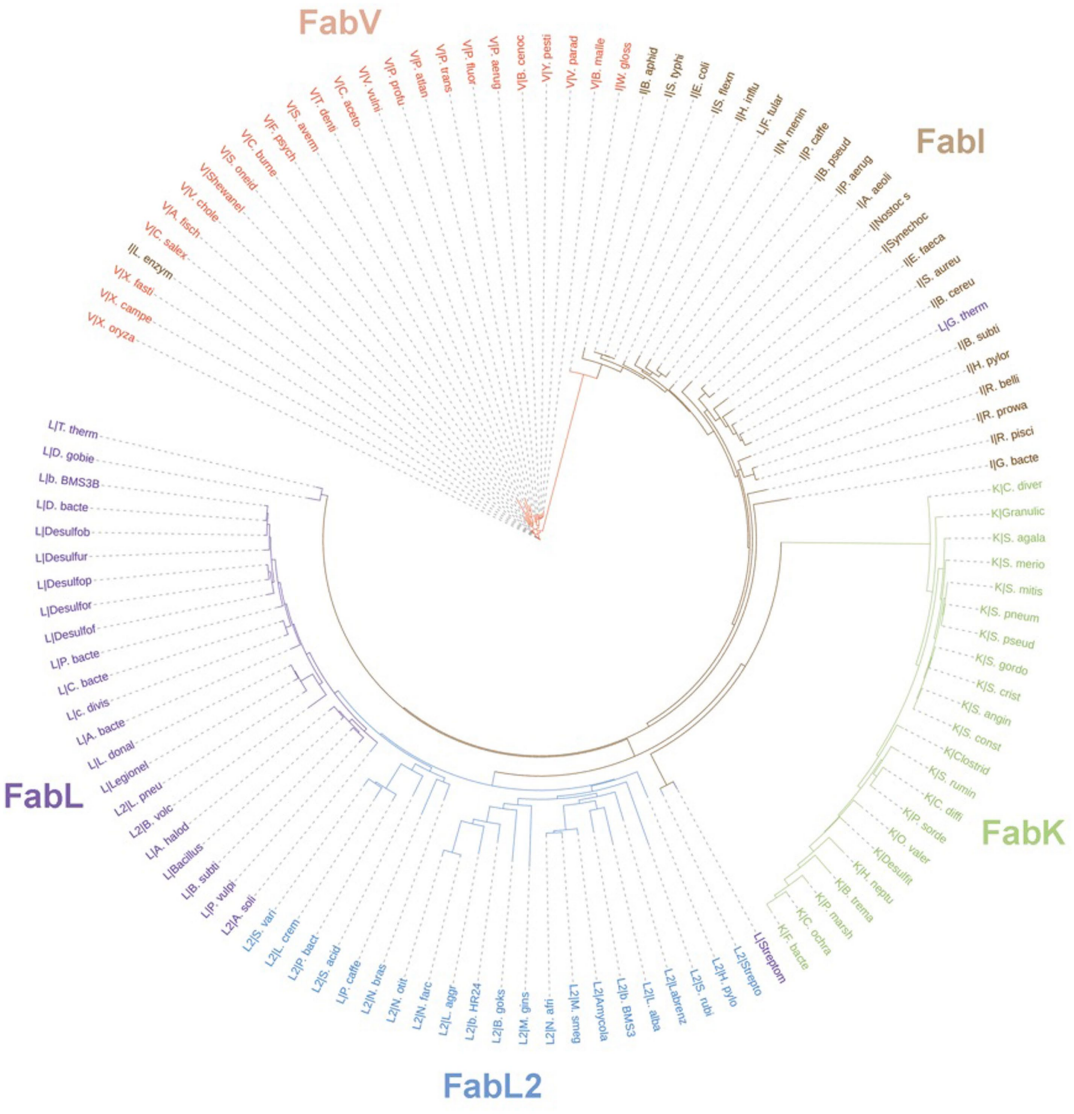
Unrooted phylogenetic tree inferred from the sequences of ENRs from the classes FabK, FabL, FabL2, FabI, and FabV. Branch lengths denote amino acid substitutions for that node. The letters behind a “|” symbol refer to their respective ENR class. The Uniprot identifiers for each one of the species present in the tree are listed on Supplementary Table 1. Sequences were downloaded from Uniprot and aligned using the multiple alignment tool ClustalW (Larkin et al., 2007). The resulting phylip alignment file was used as input for RAxML (Stamatakis, 2014) and the best substitution model was chosen automatically. Trees were visualized and customized in the webserver of iTOL (Letunic and Bork, 2021).

In prokaryotes, aside from the Archaea domain, where FA synthesis pathways seem to be ACP-independent (Lombard et al., 2012), Enoyl-ACP reductase (ENR) orthologs are widespread across the genomic landscape of most bacteria. The first one to be identified, FabI, was thought to be the only bacterial ENR, due to it being widely distributed among bacteria, until the discovery of FabL in *B. subtilis* (Heath et al., 2000c) and FabK in *S. pneumoniae* (Marrakchi et al., 2003). While FabI and FabL origins are related, FabK is structurally related to a 2-nitropropane dioxygenase (NPD). FabV, a novel ENR reported in 2008 (Massengo-Tiassé and Cronan, 2008) shows low sequence identity to both FabI and FabL and, while it belongs to the same superfamily as them, it constitutes a new ENR class. FabV was also discovered in other bacteria afterward, such as *B. mallei* (Lu and Tonge, 2010) and *P. aeruginosa* (Zhu et al., 2010). In fact, more than 3,800 FabVs are deposited on NCBI RefSeq already. ENRs diversity and phylogeny are shown in the tree depicted in Figure 13, along with FabI, FabL, FabL2, and FabK. Some sequences were grouped within an ENR class different from its annotated one, which could be due to annotation errors.

In contrast to bacteria, eukaryotic fatty acid biosynthesis is catalyzed by a type I fatty acid system, where different enzyme activities are performed by the domains of a large polypeptide (FAS-I; Smith et al., 2003), aside from plants, which use the FAS-II system, just like bacteria. Eukaryotes have two considerably distinct type I FAS: the metazoan (Maier et al., 2006) and the fungal FAS (Jenni et al., 2006). The former is a 540 kDa homodimer comprising two sets of functional domains, whose structure is common to those of bacterial polyketide synthases (Smith and Tsai, 2007). Fungal FAS form a 2.6 megadalton structure with 48 domains, and may contain a phosphopantetheinyl transferase domain, necessary for attachment to ACP (Smith and Tsai, 2007; Grininger, 2014). Evolutionary origins for this fungal FAS can be traced back to the bacterial ENRs, as it seems to share a common ancestrality with FabK from *S. pneumoniae* and other bacterial ENRs, while forming a monophyletic sister group to CMN-FAS enoyl-reductases, which are common across the groups of *Corynebacteria*, *Mycobacteria* and *Nocardia* (Bukhari et al., 2014).

Even though FAS-I systems are the most common among eukaryotes, a recent study presents phylogenetic evidence of an ENR present in bacteria, lower eukaryotes from the group Apicomplexa, and some higher eukaryotes as well (Khan et al., 2019). This protein, designated as FabI2 due to its high sequence similarity with FabI, was identified during a soil metagenomics analysis (Khan et al., 2016), and seems to be predominant in Apicomplexa and intracellular pathogenic bacteria. These novel ENRs shared by both bacteria and eukaryotes can be traced back to a common ancestral ENR closely related to FabI (Khan et al., 2019).

Domains for the SDR superfamily are found within the sequences of FabI, FabI2, and FabL. While sharing a common Rossmann fold with other SDR ENRs, a search on the Conserved Domains Database shows that FabV contains its own domain: PRK13656. The novel FabI2, spread from bacteria to higher eukaryotes (Khan et al., 2019), also contains a domain for the Rossmann-fold NAD(P)(+)-binding protein superfamily, just like its FabI counterpart. This protein, obtained from metagenomic studies, despite sharing a relatively high homology and some structural similarity with FabI, contains a unique Yx_7_K catalytic signature motif, which was thought to be specific to higher eukaryotes with plant origins (Massengo-Tiassé and Cronan, 2009; Khan et al., 2019). The most common catalytic signature among members of the SDR superfamily is Asn-x_n_-Ser-x_10_-Tyr-x_3_-Lys. In *M. tuberculosis,* the catalytic signature of its FabI, which is annotated as InhA, is Phe-x_8_-Tyr-x_6_-Lys. These catalytic signatures vary considerably amidst members of the SDR superfamily and, while higher eukaryotes such as plants are also part of it, they contain a unique signature (Thr-x_10_-Tyr-x_7_-Lys) as well (Massengo-Tiassé and Cronan, 2009).

The evolution of mycobacterial ENRs is particularly interesting, in the sense that the genome of Mtb seems to encode more than 250 enzymes involved in the metabolism of fatty acids, a vast amount when compared to the 50 contained within the genome of *E. coli* (Cole et al., 1998), despite the size similarities between these two genomes. In Mtb, though, fatty acid metabolism is vital to allow survival of the bacterium while inside its host, as its cell wall contains a lipid layer derived from the production of mycolic acids which, in turn, result from elongated fatty acids, thus justifying the presence of such a wide array of FAS enzymes (Kinsella et al., 2003). The three main explanations for this are either the birth of genes, horizontal gene transfer, or gene duplication resulting in paralogues. Mtb also has one of the highest numbers of interkingdom gene fusions—19 Mtb genes have been proposed to have a eukaryotic origin (Wolf et al., 2000; Gamieldien et al., 2002). The mycobacterial InhA, though, does not show clear evidence of horizontal gene transfer, and there are few indications of paralogy across members of its family. The enzyme also does not seem to be the result of positive selection (Kinsella et al., 2003).

## Conclusion

For many years, the catalysis of the last step of FA elongation, an essential process for almost all life forms, was thought to be performed solely by the canonical FabI in bacteria. However, in the last 20 years, we have witnessed the discovery of an amazing diversity of families of ENRs, some of them structurally and mechanistically unrelated with others. Some of these newly discovered ENR types differ in terms of structure, cofactor preferences, sensitivity to antimicrobials, in particular TCL, and kinetic mechanisms. Aside the differential susceptibilities conferred to antimicrobials and biocides, it is not clear at present the biological implications of this unexpected diversity of ENR types. However, the identification of many organisms containing more than one ENR from structurally unrelated types suggests a complex evolutionary history. In practical terms, the ever-growing list of ENRs is a treasure trove of novel opportunities for drug discovery and development.

## Author Contributions

FH: conceptualization, investigation, writing—original draft, writing review and edit. CR: investigation, writing—original draft. ES: investigation, writing—original draft. LG: investigation, writing—original draft. AC: investigation, writing—original draft. PM: resources, supervision. LB: resources, supervision. CB: conceptualization, investigation, writing—original draft, writing review and edit, resources, supervision. All authors contributed to the article and approved the submitted version.

## Funding

This study was funded by National Institute of Science and Technology on Tuberculosis (INCT-TB), Brazil (Grant numbers: 489 421703–2017-2/17–1265-8/14.2.0914.1). CB (310344/2016–6), PM (305203/2018–5) and LB (520182/99–5) are research career awardees of the National Council for Scientific and Technological Development of Brazil (CNPq). This study was financed in part by the Coordenação de Aperfeiçoamento de Pessoal de Nível Superior—Brasil (CAPES)—Finance Code 001.

## Conflict of Interest

The authors declare that the research was conducted in the absence of any commercial or financial relationships that could be construed as a potential conflict of interest.

## Publisher’s Note

All claims expressed in this article are solely those of the authors and do not necessarily represent those of their affiliated organizations, or those of the publisher, the editors and the reviewers. Any product that may be evaluated in this article, or claim that may be made by its manufacturer, is not guaranteed or endorsed by the publisher.
